# Nanowire Waveguides and Lasers: Advances and Opportunities in Photonic Circuits

**DOI:** 10.3389/fchem.2020.613504

**Published:** 2021-01-08

**Authors:** Zhiyuan Gu, Qinghai Song, Shumin Xiao

**Affiliations:** ^1^Department of Physics and Optoelectronics, Taiyuan University of Technology, Taiyuan, China; ^2^Ministry of Industry and Information Technology Key Lab of Micro–Nano Optoelectronic Information System, Shenzhen Graduate School, Harbin Institute of Technology, Shenzhen, China

**Keywords:** nanowire, lasers, cavity, photonic circuits, waveguides

## Abstract

Due to their single-crystalline structures, comparatively large aspect ratios, tight optical confinement and smooth surfaces, nanowires have increasingly attracted research interests for both fundamental studies and technological applications in on-chip photonic devices. This class of nanostructures typically have cross-sections of 2~200 nm and lengths upwards of several micrometers, allowing for the bridging of the nanoscopic and macroscopic world. In particular, the lasing behaviors can be established from a nanowire resonator with positive feedback via end-facet reflection, making the nanowire a promising candidate in the next generation of optoelectronics. Consequently, versatile nanowire-based devices ranging from nanoscale coherent lasers, optical sensors, waveguides, optical switching, and photonic networks have been proposed and experimentally demonstrated in the past decade. In this article, significant progresses in the nanowire fabrication, lasers, circuits, and devices are reviewed. First, we focus on the achievements of nanowire synthesis and introduce the basics of nanowire optics. Following the cavity configurations and mode categories, then the different light sources consisting of nanowires are presented. Next, we review the recent progress and current status of functional nanowire devices. Finally, we offer our perspective of nanowires regarding their challenges and future opportunities in photonic circuits.

## Introduction

Manipulating photons on a micro-/nano-scale has been one of the pivotal topics in photonics. For the past few decades, worldwide efforts have been devoted to explore new optical materials and structures that could confine light into the regime <100 nm or beyond the diffraction limits (Bawendi et al., 1990; Takahara et al., 1997; Barnes et al., 2003; Maier et al., 2003; Katagiri et al., 2004; Silveirinha and Engheta, 2006; Oulton et al., 2008, 2009). The pursuit of downsizing the dimensions of optical nanostructures propels the continuing success of photonic technologies for higher flexibilities, better performances, and higher-density integrations (Luo et al., 2014; Mrejen et al., 2015; Gupta et al., 2018; Gatdula et al., 2019). The intensive research on nanomaterials has led to a rich collection of nanostructures of which the shape, size, and composition can be expediently tuned. Nanostructures with fascinating material and optical properties significantly facilitate the trend of optoelectronic devices to low cost and low power consumption which could have remarkable impact in future photonics. Since the 1990s, an important class of nanostructures with cross-sections of 2–200 nm and lengths upwards of several micrometers have emerged that bridge the nanoscopic and microscopic world (Yazawa et al., 1991). They were initially called “nanowhiskers” and later “nanowires” (Xia et al., 2003). Such one-dimensional (1D) nanowires provide new opportunities and platforms that could downsize currently existing structures into subwavelength scales.

Nanowires are different from other structures such as spherical nanocrystals and quantum dots by their morphology as well as physical features. The two-dimensional light confinement allows electrons, holes, or photons to propagate freely along the third dimension. Due to their large refractive indices and smooth surfaces, the two end-facets of the nanowires could serve as mirrors to recycle the energy and pick out light with specific optical frequencies, naturally establishing the optical cavities and indicating promising applications in nanolasers. Furthermore, the subwavelength-diameter nanowires also enable light propagation with small loss and can enhance light-matter interaction results from the evanescent coupling with surroundings. Although significant progress on the fabrication technologies has been made for achieving nanostructures at a nanometer scale (Zhang et al., 2012; Cheon et al., 2017; Cho et al., 2019; Gao et al., 2020), nanowires synthesized with bottom-up growth still show natural advantages such as being defect-free and single-crystalline (Dasgupta et al., 2014). The unique geometric and material properties of nanowires are therefore quite attractive for achieving integrated light sources and many other applications such as data transmission (Ainsworth et al., 2018), sensing (Ambhorkar et al., 2018), and imaging (Park and Crozier, 2013).

The purpose of this review is to give a general overview of the nanowire fabrication, as well as review the principles of the nanowire waveguides and resonators. Then we present the recent progress on the nanowire lasers categorized by their versatile configurations and emission wavelengths, for instance, Fabry-Perot (F-P) lasers, whispering gallery mode (WGM) lasers, and single-mode lasers. Furthermore, the applications of nanowire laser are briefly discussed. Finally, we offer our perspective of nanowires regarding their challenges and future research directions.

## Nanowire Fabrication

**Figure 1:**
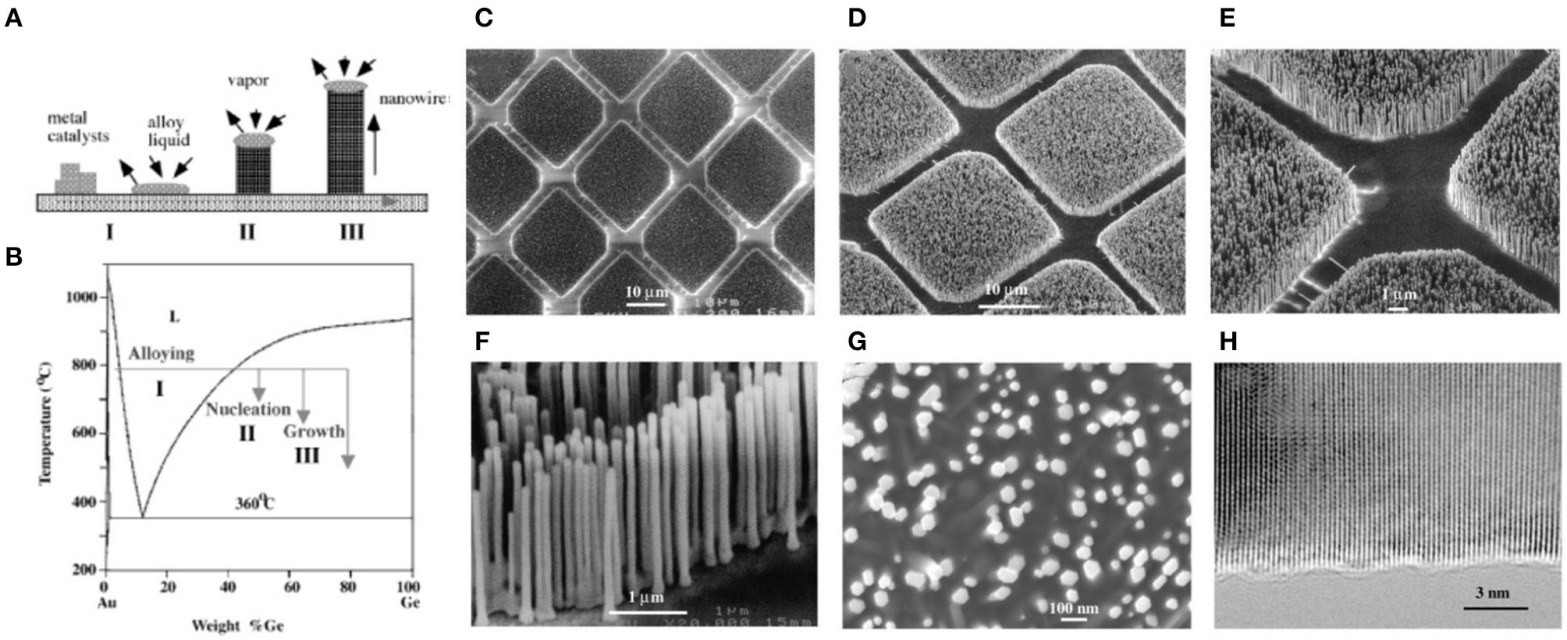
**(A)** Three stages of the vapor-liquid-solid nanowire growth mechanism. The three stages are projected onto the conventional Au-Ge binary phase diagram **(B)** to show the compositional and phase evolution during the nanowire growth process. **(C–G)** Scanning electron microscope (SEM) images of ZnO nanowires grown on sapphire substrates. **(H)** High-resolution transmission electron microscope image of an individual ZnO nanowire showing its <0001>growth direction. Scale bar **(C–H)**: 10, 10, 1, 1, 100, and 3 nm. **(A,B)** Reproduced with permission. Wu and Yang (2001) Copyright 2001, American Chemical Society. **(C–H)** Reproduced with permission. Huang et al. (2001) Copyright 2001, American Association for the Advancement of Science.

Over the past decade, many efforts have been devoted to precisely controlling the nanowire dimensions, crystal structure, composition, and growth pattern at the atomic level. The synthesis and fabrication technologies of semiconductor nanowires developed rapidly and vastly advanced the development of nanowire based optoelectronic devices (Duan et al., 2001; Harter et al., 2018). The first semiconductor nanowire was fabricated by bottom-up chemical growth from silicon using a gold metal catalyst by Wagner et al. at Bell Laboratories in the 1960s (Wagner and Ellis, 1964). This “vapor-liquid-solid” (VLS) mechanism has since been developed and extended by other researchers such as Lieber (Yang and Lieber, 1996; Morales and Lieber, 1998), Yang (Wu and Yang, 2001), and many other groups (Björk et al., 2002). The phase diagram in Figure 1A clearly shows the growth process with the VLS method. Three stages of the basic VLS growth process are included: alloying (I); nucleation (II); growth from nanorods to nanowires (III) (Wu and Yang, 2001), as shown in Figure 1B. Using the VLS growth by introducing a catalytic liquid alloy phase, various semiconductor nanowires with precise control over length, diameter, growth direction, and morphology have been successfully realized (Kuykendall et al., 2004). Typically, nanowire diameter is decided by the sizes of the metal alloy droplets. In this sense, uniform size of the nanowire arrays can be facilely achieved by employing monodispersed metal nanoparticles (Hochbaum et al., 2005). With this strategy, the length of the nanowire can be tuned from micrometer to millimeter. Furthermore, to obtain high-quality nanowire arrays, VLS epitaxy techniques have been utilized to control the orientation during the nanowire growth process (He et al., 2005). For instance, the (0001) plane of the ZnO nanowire and the (110) plane of the sapphire substrate have the good epitaxial interface, leading to the uniquely vertical epitaxial growth of the nanowires from substrate (Figures 1C–H) (Huang et al., 2001). To date, a wide range of inorganic semiconductor nanowires can be synthesized via VLS method, including group IV (Si, Ge) (Morales and Lieber, 1998; Cui et al., 2000; Wu and Yang, 2000; Sivakov et al., 2006; Colli et al., 2007), II-VI and III–V (ZnO, ZnS, CdS, CdSe, GaN, GaSb, GaAs, InP) (Duan and Lieber, 2000a,b; Zhang et al., 2001; Yang et al., 2002; Heo et al., 2004; Hsu and Lu, 2005; Noborisaka et al., 2005; Venugopal et al., 2005; Moore and Wang, 2006; Shtrikman et al., 2009; Zhai et al., 2010; Zhao et al., 2012), and alloy nanowires (CdSSe, ZnCdS, ZnCdSSe) (Duan and Lieber, 2000a; Radovanovic et al., 2005; Johansson et al., 2006; Kuykendall et al., 2007; Mårtensson et al., 2007; Pan et al., 2009a; Gu et al., 2011; Yang et al., 2011).

## Nanowire Fundamentals

### Mechanisms for nanowire cavity

**Figure 2:**
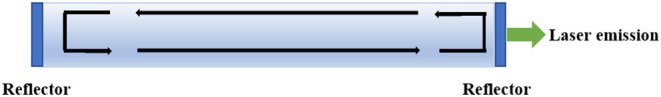
Schematic of nanowire F-P cavity.

To establish lasing, three essential factors must be satisfied: (1) gain medium for population inversion; (2) pump source for exciting medium; (3) optical cavity for amplifying light. Usually, nanowire itself can serve as a gain medium. With the excitation of external light sources, population inversion can be achieved. The end-facets of nanowire naturally play the roles of reflection mirrors due to large index differences with its surroundings, satisfying the conditions to form an optical cavity, as shown in Figure 2. The light in the nanowire cavity can be amplified by optical feedback and thus lasing can be obtained when the round-trip gain compensates the total cavity losses,

(1) $$\begin{array}{r} \Gamma g>\alpha_{m}+\alpha_{p}=\frac{1}{2L}\ln\left(\frac{1}{R_{1}R_{2}}\right)+\alpha_{p} \end{array}$$

where Γ is the confinement factor, *g* is the material gain, α_*m*_ is the mirror loss, α_*p*_ is the propagation loss, *L* is the cavity length, and *R*_1_, *R*_2_ are the effective reflection coefficients of the two end-facets. Therefore, from Equation (1), the key points for achieving low-threshold lasing are to increase the gain efficiency *g* or to reduce cavity loss. Furthermore, the estimation of the reflectivity for the guided modes in nanowire is complicated because their diameter is typically smaller than the lasing wavelength that leads to the diffraction effect at the nanowire edges. For a nanowire vertically standing on a substrate (Huang et al., 2001), the ends of reflectivity at the two facets are different (Maslov and Ning, 2004). The reflection coefficient from the top facet grows with the mode confinement as well the frequency. However, the reflection coefficient from the bottom facet does not grow monotonically with mode confinement, which does not match the prediction from the Fresnel formula. The reason is that the optical fields outside of the nanowire experience a jump in the dielectric constant, resulting in larger reflectivity at low frequencies despite the small refractive index contrast between nanowire and substrate.

The research on nanowire lasers increases apace, so rigorous modeling of nanoscale lasers, therefore, becomes increasingly important. The coupled rate equations to describe the carrier density and photon density for a single mode in a semiconductor cavity is an effective approach to analyze the lasing threshold, which can be defined as

(2) $$\begin{array}{r} \frac{dN}{dt}=\eta P-\frac{N}{\tau_{r}}-\frac{N}{\tau_{nr}}-\Gamma \nu_{g}\alpha \left(N-N_{0}\right)S \end{array}$$

(3) $$\begin{array}{r} \frac{dN}{dt}=\beta \frac{N}{\tau_{r}}+\Gamma \nu_{g}\alpha \left(N-N_{0}\right)S-\frac{1}{\tau_{S}}S \end{array}$$

where *N* is the carrier density, *S* is the photon density, *P* is the pump intensity, η is the pumping efficiency, τ_*r*_ and τ_*nr*_ are the spontaneous emission and non-radiative lifetime, respectively, τ_*S*_ is the photon lifetime, β is the spontaneous emission factor, *N*_0_ is the transparency carrier density, α is the differential gain, *?* is the confinement factor and ν_*g*_ is the group velocity. By solving for these coupled rate equations under steady-state conditions, the photon density can be plotted as a function of pump intensity (Oulton et al., 2009). Then the lasing threshold can be more quantitatively defined by fitting the experimental power plot to a calculated plot.

Another key figure of merit for nanowire lasers is the confinement factor Γ. Γ represents the overlap between the resonant mode and gain medium. In this sense, the nanowire size and shape as well as the mode profile are pivotal parameters that determine Γ. For example, a confinement factor of >0.8 can be obtained for the nanowire with a sufficiently large triangular cross section (Seo et al., 2008). For typical semiconductor nanowires formed with wurtzite crystal structures such as GaN, CdS, and ZnO, their geometrical axis coincides with the optical axis of the crystal (also called the -axis) (Maslov and Ning, 2004). The optical gain in wurtzite bulk crystals depends on the polarization of the lasing mode. Therefore, the orientation of the electric field of the mode in nanowire relative to the optical axis of the crystal is essential to determine the modal gain. According to the polarization of optical modes in nanowire, one can introduce the longitudinal gain *G*_∥_, transverse gain *G*_⊥_, longitudinal confinement factor Γ_∥_, and transverse confinement factor Γ_⊥_. The total gain of mode can be written as

(4) $$\begin{array}{r} G=\Gamma_{∥}G_{∥}+\Gamma_{⊥}G_{⊥}. \end{array}$$

### Mechanisms for Nanowire Waveguide

The theories about waveguiding and light confinement have been extensively studied in freestanding nanowire in the visible region (Maslov and Ning, 2004; Eaton et al., 2016). The ability of nanowire structure to support multiple modes has been confirmed by solving Maxwell's equations analytically. For a circular-cross-section nanowire, the modes can be analytically solved in cylindrical coordinates with the following eigenvalue equations (Ma et al., 2013):

TE_0m_ modes

(5) $$\begin{array}{r} \frac{J_{\nu }^{\prime }\left(U\right)}{UJ_{\nu }\left(U\right)}+\frac{K_{\nu }^{\prime }\left(W\right)}{WK_{\nu }\left(W\right)}=0, \end{array}$$

TM_0m_ modes

(6) $$\begin{array}{r} \frac{J_{\nu }^{\prime }\left(U\right)}{UJ_{\nu }\left(U\right)}+\frac{n_{2}^{2}K_{\nu }^{\prime }\left(W\right)}{n_{1}^{2}WK_{\nu }\left(W\right)}=0, \end{array}$$

**Figure 3:**
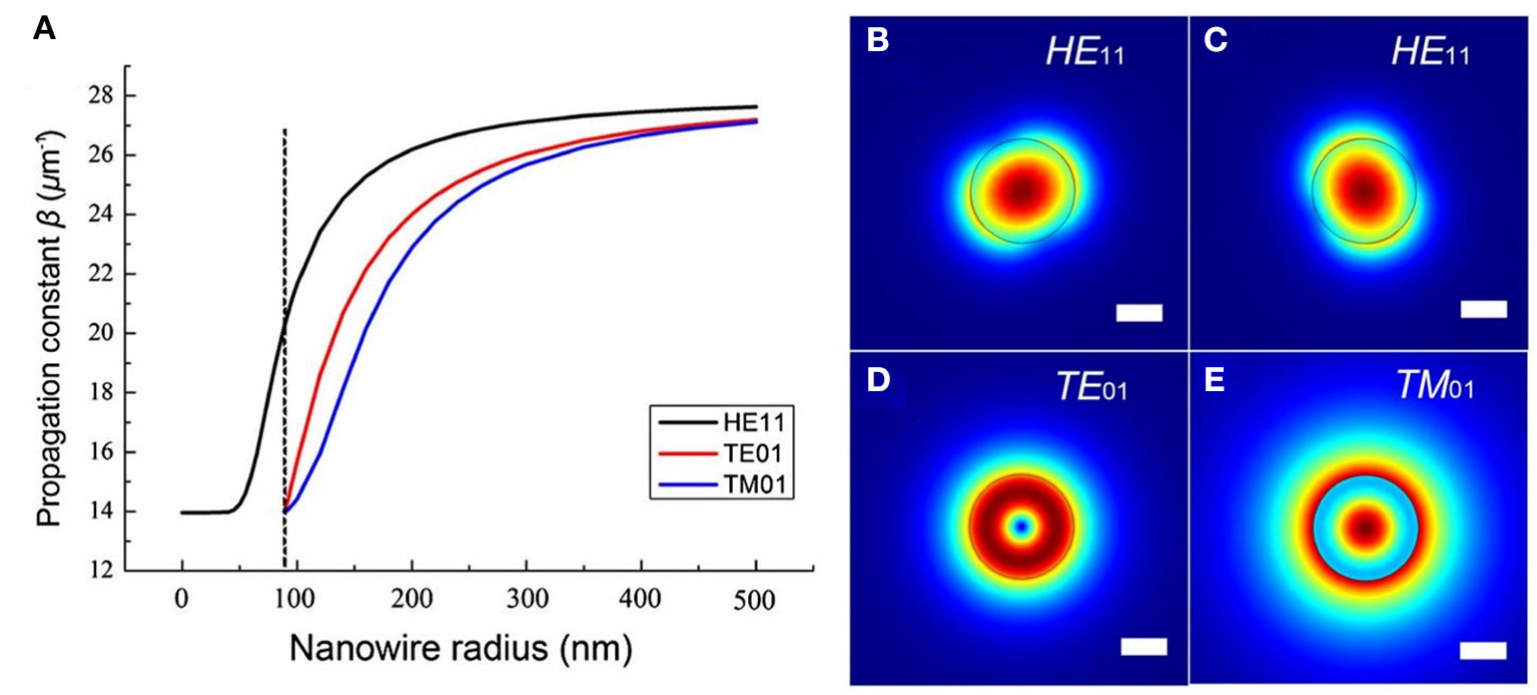
**(A)** The propagation constant β of the HE_11_, TM_01_, and TE_01_ modes as a function of ZnO nanowire radius. Dashed line represents the single-mode condition. **(B)** Electric field distributions of the HE_11_, TM_01_, and TE_01_ modes for ZnO nanowire with 240 nm diameter at a wavelength of 420 nm. Scale bar: 100 nm. **(A–E)** Reproduced with permission. Ma et al. (2013) Copyright 2013, Optical Society of America.

HE and EH modes

(7) $$\begin{array}{r} \left\{\frac{J_{\nu }^{\prime }\left(U\right)}{UJ_{\nu }\left(U\right)}+\frac{K_{\nu }^{\prime }\left(W\right)}{WK_{\nu }\left(W\right)}\right\}\left\{\frac{J_{\nu }^{\prime }\left(U\right)}{UJ_{\nu }\left(U\right)}+\frac{n_{2}^{2}K_{\nu }^{\prime }\left(W\right)}{n_{1}^{2}WK_{\nu }\left(W\right)}\right\}\\ ={\left(\frac{\nu \beta }{kn_{1}}\right)}^{2}{\left(\frac{V}{UW}\right)}^{4}, \end{array}$$

where *J*_ν_ is the Bessel function of the first kind, and *K*_ν_ is the modified Bessel function of the second kind. $U=a{\left(k_{0}^{2}n_{1}^{2}-\beta^{2}\right)}^{\frac{1}{2}}$, $W=a{\left({\beta^{2}-k}_{0}^{2}n_{2}^{2}\right)}^{\frac{1}{2}}$, and $V=ak_{0}{\left(n_{1}^{2}-n_{2}^{2}\right)}^{\frac{1}{2}}$ are waveguide parameters, *n*_1_, *n*_2_ are refractive indices of the nanowire and the surrounding medium, *a* is the radius of the nanowire, $k=\frac{2\pi }{\lambda }$, λ is the wavelength of light in vacuum, and β is the propagation constant. By numerically solving Equations (5–7), waveguiding modes supported by the nanowire can be obtained. For reference, Figure 3A gives the evolution of the propagation constant β of the fundamental modes at different nanowire radii (Ma et al., 2013). At higher diameters, several modes with strong confinement are sustained within the nanowire. Though the propagation loss is reduced, the multiple mode regime will spoil the device performance that leads to mode competition. Furthermore, the free-standing nanowire usually exhibits tightly confined electromagnetic fields with a high fraction of evanescent fields, as shown in Figures 3B–E. Interestingly, when the nanowire diameter is small enough, the fundamental transverse mode TE_01_ and TM_01_ can be suppressed and single-mode operation is available. Furthermore, the effective index of the survived mode, associated with propagation velocity, is very close to the air, such that the evanescent wave outside the nanowire is considerably large, undoubtedly resulting in a small confinement factor and thus less overlap with the gain medium.

## Nanowire Lasers

The large refractive index difference between the semiconductor material and its surrounding dielectric environment enables light confinement in the nanowire. Thus, the two end-facets function well as the reflection mirrors and constitute the optical cavities for light amplification. Benefitting by the capability of emission-wavelength tuning, complementary metal-oxide-semiconductor (CMOS) compatibility, and miniaturization, the semiconductor nanowires have been widely recognized as promising candidates for significant building blocks for the next-generation of integrated photonics. In the past few decades, many types of semiconductor nanowires have been fabricated and thoroughly investigated. Meanwhile, considering the less trap-state density, the single-crystalline nanowire enabled F-P cavities usually exhibit good performance such as low lasing threshold. In this section, we introduce the typical nanowire lasers including photonic lasers, plasmonic lasers, single-mode lasers, and wavelength-tunable lasers.

### Fabry-Perot Lasers

#### Photonic Lasers

**Figure 4:**
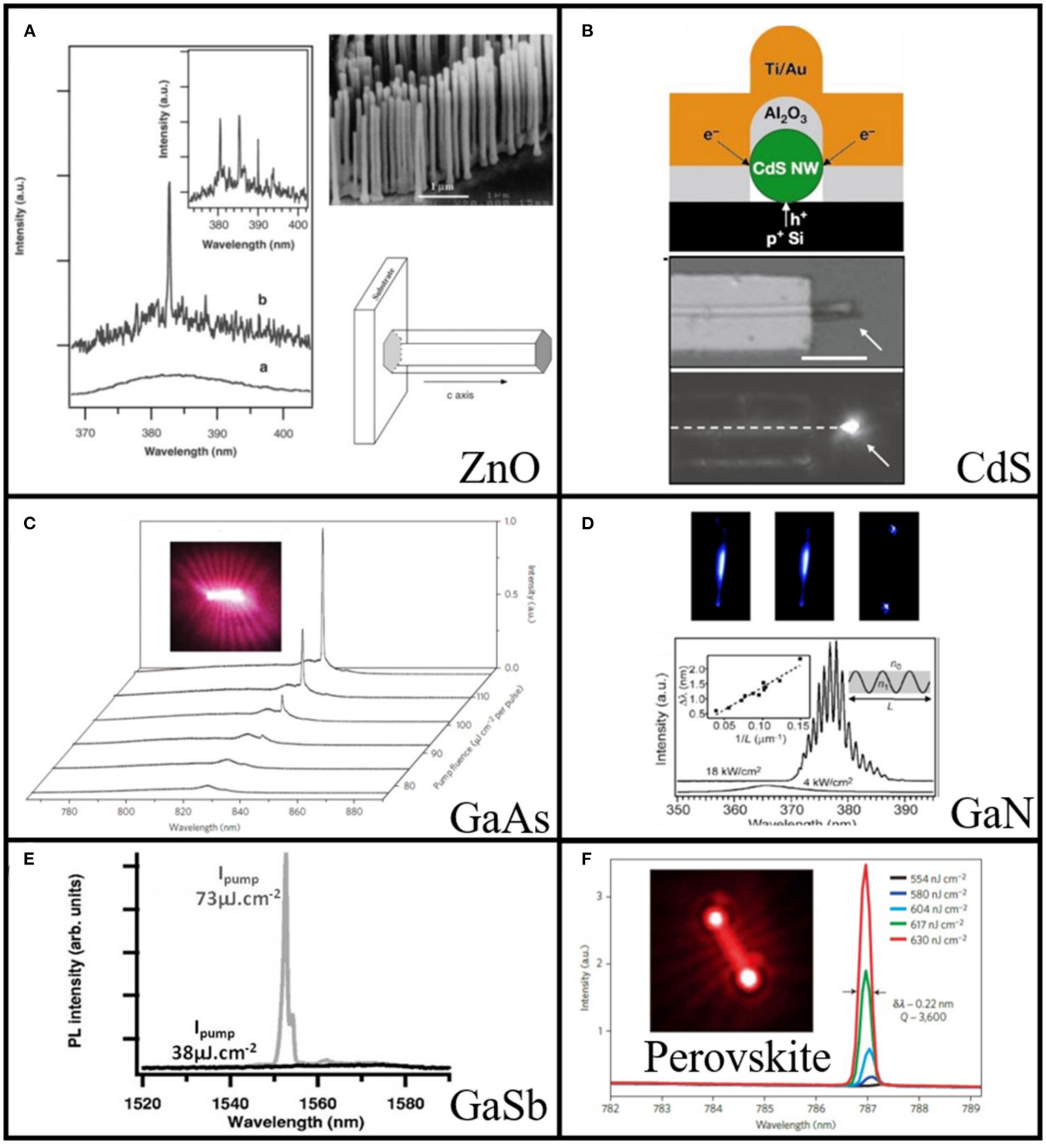
**(A)** Emission spectra from ZnO nanowire arrays below (line a) and above (line b and inset) the lasing threshold. The pump power for these spectra are 20, 100, and 150 kW cm^−2^, respectively. Upper right panel: the SEM image of the as-synthesized ZnO nanowire, scale bar:1 μm. Lower right panel: schematic illustration of a nanowire as a resonance cavity with two naturally faceted hexagonal end faces acting as reflecting mirrors. **(B)** Upper panel: schematic of electrically driven nanowire lasers made of CdS. Lower panel: SEM image and electroluminescence image of the device. Scale bar: 5 μm. **(C)** GaAs nanowire emission spectra with increasing pump fluence around threshold and optical image of nanowire above threshold (inset). **(D)** Upper panel: optical images of the single GaN nanowire at 4, 17, and 66 kW cm^−2^ (from left to right). Lower panel: PL spectra of the GaN nanowire under excitation of 4 and 18 kW cm^−2^. Left inset: mode spacing as a function of inverse nanowire length. Right inset: schematic representation of a longitudinal nanowire cavity. **(E)** PL spectra of GaSb nanowire laser below (38 μJ cm^−2^) and above (73 μJ cm^−2^) threshold. **(F)** Spectra evolution of single MAPbI_3_ nanowire around lasing threshold. Inset shows the fluorescence image above threshold. **(A)** Reproduced with permission. Huang et al. (2001) Copyright 2001, American Association for the Advancement of Science. **(B)** Reproduced with permission. Duan et al. (2003) Copyright 2003, Nature Publishing Group. **(C)** Reproduced with permission. Saxena et al. (2013) Copyright 2013, Nature Publishing Group. **(D)** Gradečak et al. (2005) Copyright 2005, American Institute of Physics. **(E)** Reproduced with permission. Chin et al. (2006) Copyright 2006, AIP Publishing LLC. **(F)** Reproduced with permission. Zhu et al. (2015) Copyright 2015, Nature Publishing Group.

In 2001, Huang et al. reported the first nanowire cavity and laser from multiple ZnO nanowires synthesized by VLS approach (Huang et al., 2001). Under the excitation of He-Cd laser (325 nm), clear near-band-gap edge emission at 377 nm has been observed, as shown in Figure 4A. By conducting the power strength-dependent pumping with the fourth harmonic of Nd: yttrium-aluminum-garnet laser (266 nm, 3 ns pulse width) at room temperature, the stimulated emission has been explored and confirmed. The lasing action in these ZnO nanowires was clearly observed without any fabricated reflection mirrors (see insert in Figure 4A) by steadily increasing the pump power, explicitly proving the formation of F-P cavity and positive feedback. Above the threshold, sharp peaks with linewidth <0.3 nm emerged in the emission spectra. The demonstration of these ZnO nanowire lasers paves the way to realize lasers in nanoscale and leads to a considerable focus of the research on nanowire geometry. Shortly after, ZnO nanolasers with external quantum efficiency (QE) as high as 60% and internal QE of 85% were reported (Zhang et al., 2005). Two- and three-photon absorption with threshold of 100 mJ cm^−2^ were also investigated in ZnO nanowires (Zhang et al., 2006). In 2009, Zhang et al. further reduced the threshold to 160 μJ cm^−2^ from two-photon absorption of ZnO nanowires (Zhang et al., 2009). Following these pioneering works, II-VI semiconductors became the focus of investigation. The CdS nanowire lasing properties under different temperatures were then reported by Lieber et al. at Harvard University (Figure 4B) (Duan et al., 2003). In 2004, Ding et al. reported the first ZnS nanowire lasers (Ding et al., 2004). Moreover, the III-V compounds with emission wavelength spanning from ultraviolet (UV) to near Infrared (IR) such as GaAs (Figure 4C) (Saxena et al., 2013), GaN (Figure 4D) (Johnson et al., 2002; Zhang et al., 2014b), InGasAs/GaAs (Chen et al., 2011), GaAs/GaAsP (Johnson et al., 2001), GaSb (Figure 4E) (Chin et al., 2006), and InGaN/GaN (Wu et al., 2011) have been successfully demonstrated.

In addition to traditional II-VI and III-V semiconductors, a new class of materials with chemical formula ABX_3_ (metal halide perovskite) have been developed in the past few years. A can be referred to as methylammonium (CH_3_NH_3_ or MA), formamidinium (CH(NH_2_)_2_ or FA), cesium (Cs), B to lead (Pb), tin (Sn), and X to halide (chloride (Cl), bromide (Br), iodide (I)). Such type of material exhibits outstanding features including high absorption coefficients, low defect densities, long carrier diffusion lengths, direct bandgap (Green et al., 2014; Sum and Mathews, 2014; Stranks and Snaith, 2015; Brenner et al., 2016; Correa-Baena et al., 2017; Ha et al., 2017; Huang et al., 2017; Zhang et al., 2017). The excellent optical and material properties of the perovskite material have attracted tremendous research interests ranging from lasers (Sutherland et al., 2014; Zhang et al., 2014a), solar cells (Kim et al., 2012; Yang et al., 2015; Anaraki et al., 2016; Bi et al., 2016), optoelectronics (Tan et al., 2014; Jaramillo-Quintero et al., 2015; Saidaminov et al., 2015), and sensing (Zhu et al., 2018; Rahimi et al., 2019). In 2015, Zhu et al. reported the perovskite nanolasers with ultralow lasing threshold in high-quality perovskite nanowires (Zhu et al., 2015). They chemically synthesized the single-crystal perovskite nanowires via a two-step solution processed surface-initiated growth approach. A single peak has been observed with the excitation of the femtosecond laser (Figure 4F). The “S-like” light-light curve confirmed the formation of lasing behavior. The lowest measured lasing threshold of the perovskite nanowire laser is as small as 220 nJ cm^−2^, which is far lower than other perovskite nanostructures (Deschler et al., 2014; Zhang et al., 2014a). Due to the high crystal quality enabled smooth surface, the full width at half maxima (FWHM) of the lasing peak is quite narrow (~0.22 nm) and later reduced to 0.08 nm (Wang et al., 2017).

#### Plasmonic lasers

The fast development of nanowire nanolasers has significantly advanced the research on nanowire photonics. Nowadays, device miniaturization in fundamental sciences and industrial applications is a long-term and continuous pursuit. Though the semiconductor nanowires could confine light in a wavelength scale, the demonstrated nanowire nanolasers are still constrained by the diffraction limit and their dimensions are larger than the half of the optical wavelength in free space. In the past decade, a new type of nanolasers, based on semiconductor nanowires and metal substrates, have attracted widespread attention for achieving deep sub-wavelength coherent light sources since their first creation in 2009 (Oulton et al., 2009). The generated surface plasmonics at the dielectric-metal interface strongly couple with the waveguide modes within the semiconductor nanowires, which were named as “hybrid surface plasmonic modes” (HSPMs). The HSPMs transfer the light to electron oscillations and propagate along the surface of the metal substrate but are simultaneously guided by the nanowire waveguide effect. As a result, the physical dimension of the optical devices can be compressed to the nanometer scale. Furthermore, the photon-plasmon interaction immensely enhances the local field in such structures, enabling the realization of plasmonic nanolasers with high power, low consumption, and fast response. In this section, we introduce and review in detail the plasmonic nanolasers including hybrid surface plasmonic lasers, photonic-plasmonic integrated lasers, and wavelength-tunable plasmonic lasers.

##### Hybrid surface plasmonic lasers

**Figure 5:**
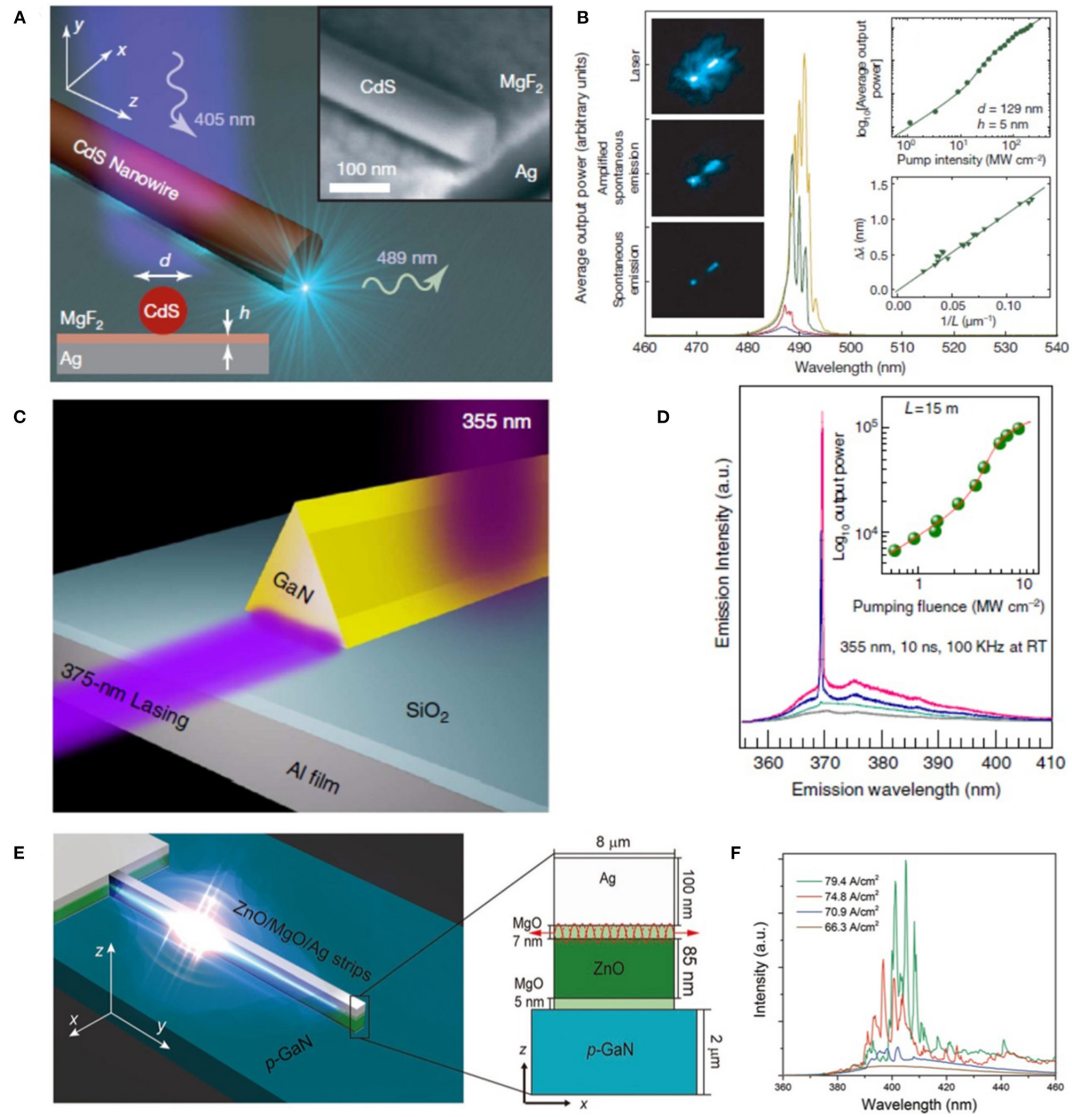
**(A)** The plasmonic laser consists of a CdS semiconductor nanowire on top of a silver substrate, separated by a nanometer-scale MgF_2_ layer of thickness *h*. Inset: SEM image of the structure. Scale bar: 100 nm. **(B)** Laser oscillation of a plasmonic laser. The four spectra for different peak pump intensities show the transition from spontaneous emission (21.25 MW cm^−2^) via amplified spontaneous emission (32.50 MW cm^−2^) to full laser oscillation (76.25 and 131.25 MW cm^−2^). Left inset: optical images at different pumping intensities. Upper right inset: The non-linear response of the output power to the peak pump intensity. Lower right inset: The relationship between mode spacing Δλ and nanowire length *L*. **(C)** Schematic of GaN plasmonic devices. **(D)** Power-dependent emission spectra of the plasmonic devices. Inset shows the light-light curve. **(E)** The schematic diagram of the p-GaN/MgO/ZnO/MgO/Ag structure. Inset illustrates the SPPs feedback diagram in the ZnO/MgO/Ag cavity. **(F)** Injection current density dependent emission spectra of the plasmonic laser. **(A,B)** Reproduced with permission. Oulton et al. (2009) Copyright 2009, Nature Publishing Group. **(C,D)** Reproduced with permission. Zhang et al. (2014b) Copyright 2014, Nature Publishing Group. **(E,F)** Reproduced with permission. Yang X. et al. (2019) Copyright 2018, Wiley-VCH.

In 2008, Oulton et al. presented the concept of a hybrid plasmonic waveguide in which a nanowire and metal substrate is separated by a thin dielectric film with a low refractive index (Oulton et al., 2008). The continuity of the displacement field of normal to substrate endows the extreme light confinement within the thin insulator layer and ultrasmall mode volume. Mode area from λ^2^/40-λ^2^/400 has been achieved in this plasmonic waveguide. They theoretically and numerically demonstrated that the hybrid surface plasmonic modes simultaneously come from strong light localization of surface plasmonic mode and low propagation loss of photonic mode. In 2009, the same group experimentally demonstrated the plasmonic lasers from a CdS nanowire, separated from a silver surface by a nanometer-thick MgF_2_ insulating gap (Figure 5A) (Oulton et al., 2009). The plasmonic device was optically pumped with a femtosecond pulsed laser (405 nm, 100 fs) below 10 K. With the increasing of the pump fluence, clear transition from spontaneous emission to lasing from the dominant I_2_ CdS exciton line at 489 nm has been observed (Thomas and Hopfield, 1962). By integrating the lasing intensities, an “S-like” curve can be obtained which matches well with the rate equation (inset of Figure 5B) and confirms the lasing action. The measured free spectral range (FSR) of the lasing modes exhibited a linear relationship with the reciprocal of the nanowire length, definitely notarizing the existence of F-P resonant modes (inset of Figure 5B). Above the threshold, bright blue spots could be seen due to the scattering at the nanowire end-facets (left inset of Figure 5B). Notably, due to the high intrinsic metal ohmic losses and radiation leakage, the proposed plasmonic lasers still need higher power to be excited compared with their photonic counterpart. However, the realization of plasmonic nanolasers from nanowire represents a significant step toward integrating devices with high-density and small footprints. After the first demonstration of hybrid surface plasmonic lasing from nanowires, the research on this type of nanolaser has since expanded rapidly and vastly boosted the development of lasers from physics to cavity design.

At the initial stage for implementing plasmonic nanowire lasers, cryogenic operation is widely utilized in the process of measuring plasmonic nanowire lasers to promote the optical gain and prevent device damages (Oulton et al., 2009; Nezhad et al., 2010; Ma et al., 2011), which is still one of the obstacles and truly limits their practical applications. In 2014, Zhang et al. reported the UV plasmonic laser devices with low-loss from triangular GaN nanowires (Zhang et al., 2014b). In this work, several strategies have been executed to reduce the lasing threshold of plasmonic lasers. First, aluminum (Al) film was chosen to be the substrate and plasmonic medium to impair the ohmic loss instead of silver substrate with high loss at UV region (Huang et al., 2001). Second, they used nanowires with triangular cross-sections to construct a fully planar semiconductor-insulator-metal interface (Figure 5C). The large contact interface between the nanowire and the nether insulator-metal ensures a sufficient photonic-plasmonic modal overlap, providing an effective channel for exciton-plasmon coupling. Last, the planar nanowire-dielectric interface suffers smaller scattering loss compared with previous works (Oulton et al., 2009; Lu et al., 2012). By adopting these improvements, a threshold of 3.5 MW cm^−2^ (Figure 5D, inset) has been obtained which is three orders of magnitude lower than that of the previously reported room temperature plasmonic nanolasers (Ma et al., 2011). Furthermore, Chou et al. designed a plasmonic nanolaser without additional insulator layers in 2016 (Chou et al., 2016). The average lasing threshold of the presented structure was around 20 MW cm^−2^, which was four-times lower than that of structures with insulator layers. The lower lasing threshold was ascribed to the relatively strong mode confinement at the Al-ZnO interface.

Usually, the as-introduced nanolasers are optically excited by femtosecond pulsed lasers which prevents the practical application of such devices. To overcome this limit, using electric injection is an alternative route to achieve amplified light emission from plasmonic lasers. However, the large ohmic losses and joule heating of electrical injection tremendously weaken the effective gain and consequently induce the increasing of operation threshold. The threshold of the demonstrated electrically driven plasmonic lasers were almost one order of magnitude larger than their photonic counterpart. Furthermore, the high injection currents may change the emission spectra and also affect the stability and reliability of plasmonic lasers (Zhang Y. et al., 2019). The first electrically driven plasmonic laser was demonstrated by Hill et al. in 2009 (Zhou et al., 2013). Super-linear emission with a line width of ~0.5 nm was observed by employing electric injection above 10^4^ A cm^−2^. Unfortunately, the proposed devices can only be operated under a cryogenic environment due to poor heat dissipation. To reduce the lasing threshold and realize room-temperature lasing for plasmonic nanolasers, Ding et al. improved the fabrication process to minimize imperfections of plasmonic devices (Ding et al., 2013). After conducting several strategies, they successfully realized the lasing of plasmonic structures at room-temperature. In 2019, Yang et al. designed and experimentally demonstrated an electrically driven UV plasmonic nanowire laser at room-temperature, realized in a p-GaN/MgO/ZnO/MgO/Ag structure, as shown in Figure 5E (Yang X. et al., 2019). The high optical gain coefficient, large exciton binding energy, large oscillator strength, and material stability of ZnO was selected to ensure the performance of the device under high current injection and beneficial for a faster radiative lifetime (Djurišić et al., 2010; Sidiropoulos et al., 2014; Chou et al., 2015) due to its facile coupling with surface plasmon polaritons (SPPs). In this configuration, the electrons were injected into ZnO layer from Ag layer through MgO gap layer, while holes were injected from p-GaN layer by tunneling effect. The MgO layers between ZnO active region and p-GaN layer was introduced to block electrons from entering into the p-GaN and enhance the emission from ZnO. As a result, an extremely low threshold of 70.2 A cm^−2^ was achieved at room temperature and the output power reached 30 μW when the injection current was 40 mA. Figure 5F summarized the relationship between injection current density and emission spectra of the device collected from the lateral direction of the cavity. At an injection current density of 66.3 A cm^−2^, a broad spontaneous emission peak was observed. Sharp lasing peaks with FWHM of 1.2 nm emerged when the injection current was improved to 70.9 A cm^−2^. Given a higher injection current (74.8 A cm^−2^), multiple modes with higher intensities were excited which corresponds to an effective index of 3.65, clearly suggesting the lasing action from the device.

##### Photonic-plasmonic integrated structures

**Figure 6:**
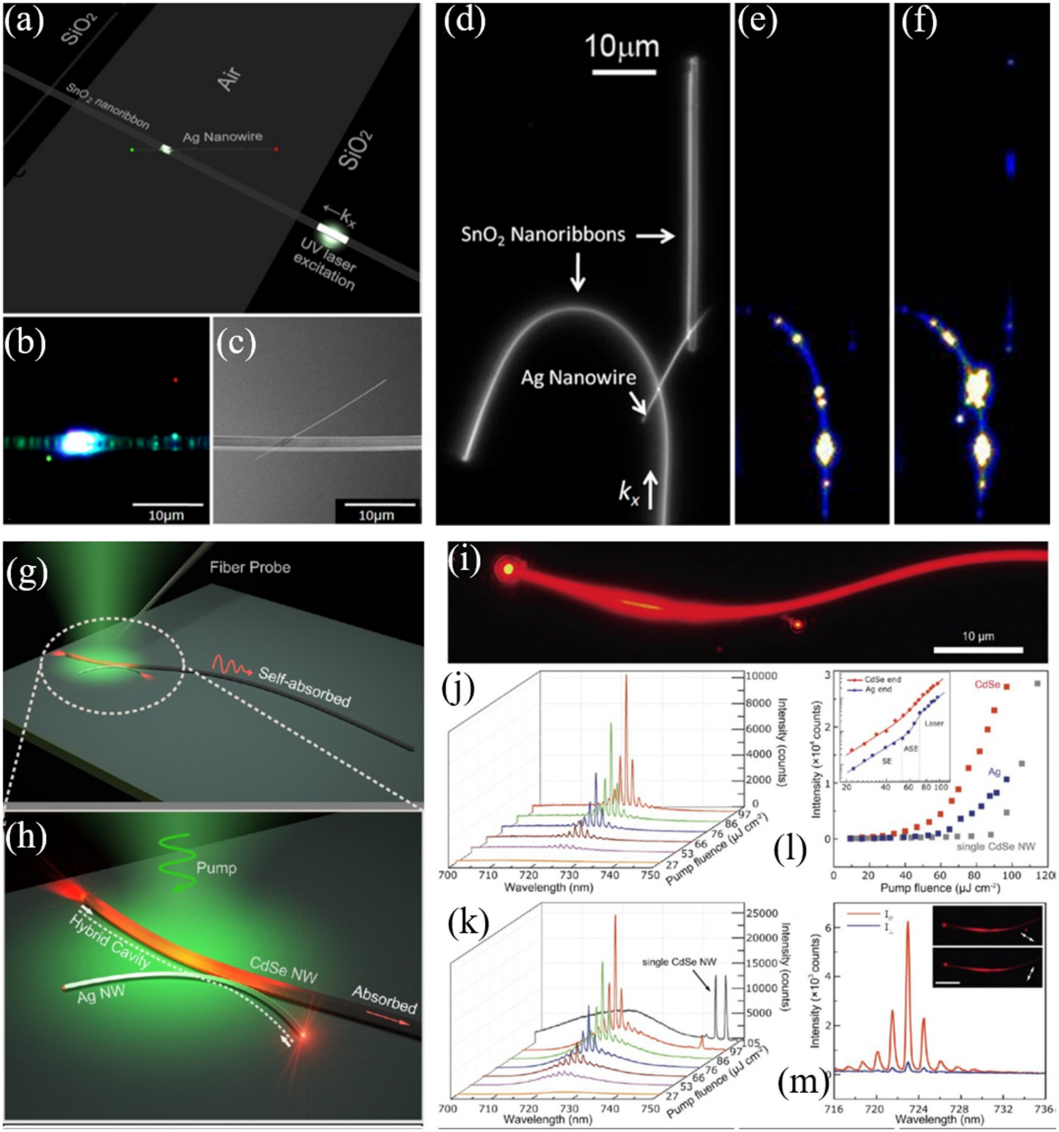
**(a)** Schematic representation of the photonic-plasmonic routing device. **(b)** Optical microscope image of the SnO_2_ waveguiding and routing. Scale bar: 10 μm. **(c)** SEM image of the device. Scale bar: 10 μm. **(d)** Dark field optical image of the SnO_2_/Ag/SnO_2_ coupling device. Scale bar: 10 μm. Optical image when the PL of the bent SnO_2_ nanoribbon was excited from the bottom end with **(e)** and without **(f)** the bridging Ag nanowire. **(g)** Schematic of the hybrid photon-plasmon nanowire laser. **(h)** Closed-up view of the coupling area. **(i)** Optical microscope image of the hybrid structure under pumping intensity 97 μJ cm^−2^. Scale bar: 10 μm. Lasing spectra collected from the Ag **(j)** and CdSe **(k)** end-facet under pump fluences of 27–97 μJ cm^−2^. **(l)** Emission intensity vs. pump fluence collected from end-facets of the CdSe nanowire (red squares) and the Ag nanowire (blue squares). The gray-square pattern is from the single CdSe nanowire without coupled Ag nanowire. Insets show the log-log scale relation between emission intensity vs. pump fluence. **(m)** Polarization-sensitive lasing spectra from Ag nanowire end-facet with the emission polarization oriented parallel (red line) and perpendicular (blue line) to the Ag nanowire. Inset: dark-field microscope images show the polarization-dependent lasing outputs. Scale bar: 10 μm. **(a–f)** Reproduced with permission. Yan et al. (2009) Copyright 2009, National Academy of Sciences. **(g–m)** Reproduced with permission. Wu et al. (2013) Copyright 2013, American Chemical Society.

SPPs represent one of the most promising candidates to break diffraction limits imposed by conventional photonic structures. Though the subwavelength–scale mode volume makes it possible to build optical devices with high-density and better performance, the large metal loss hinders the possibility to channel information across the entire plasmonic waveguides. Hence, the optical routing by integrating metal and dielectric nanowires is a significant issue to be addressed. Furthermore, the excitation of SPPs faces a severe challenge due to the momentum mismatch between SSPs and photons. In 2009, Yan et al. proposed a simple approach to couple dielectric mode in SnO_2_ nanoribbon with SPPs in silver nanowires (see Figure 6a) (Yan et al., 2009). A silver nanowire was placed on the top of a SnO_2_ nanoribbon waveguide that bridges two SiO_2_ substrates, as the SEM image shown in Figures 6c,d. The photoluminescence (PL) was excited by a UV laser with a focused spot on the lower right of the SnO_2_ nanoribbon. The photons propagating in the SnO_2_ waveguide were scattered at the metal-dielectric contact point that provides wave vectors in a broad range and compensates the momentum mismatch of photons and SPPs. Then the excited SPPs traveled along the silver nanowire and emitted into free space at the end of the wire, as shown in Figure 6b. It is noted that the SPP propagation loss was related to the frequency. The high frequency suffers larger loss than the low frequency, so the frequency distribution of the SSPs was expected to red shift which accounts for the red spot on the upper right end of the silver nanowire (Figure 6b). Furthermore, the dielectric-metal-dielectric coupling device was designed (see Figures 6d–f). Two SnO_2_ nanoribbons bridged by a silver nanowire were placed on the substrate. When the bent nanoribbon was excited, the guided light propagated along the nanoribbon. As expected, the light was scattered and then coupled into silver wire at the left SnO_2_-Ag joint point. The excited SPPs consequently propagate to the straight ribbon without any excitation, distinctly confirming the coupling between SPPs and photons. For comparison, the case of without silver nanowire was also conducted and no signals were detected from the straight ribbon. This work proves the possibility of incorporating metal nanowire in photonic circuits and can be extended to other dielectrics such as Si, SiO_2_, and GaN.

For hybrid plasmonic structures, one of the disadvantages is the mixture property of plasmonic and photonic components, making it difficult to extract a pure plasmonic mode from the generated mode (Hill et al., 2007; Noginov et al., 2009; Oulton et al., 2009; De Leon and Berini, 2010; Gather et al., 2010; Ma et al., 2011; Lu et al., 2012; Liu N. et al., 2013). In 2013, Wu et al. attempted to solve this issue by designing a new cavity scheme (Wu et al., 2013). As shown in Figures 6g,h, a bent CdSe nanowire was placed on the substrate and then a curved silver nanowire was coupled to this CdSe nanowire with a small point. When the semiconductor CdSe nanowire was optically pumped, the longitudinal photonic modes could be excited and then propagated along the CdSe nanowire. Consequently, the guiding modes from CdSe nanowire effectively excited the SPP waves in the silver nanowire. Importantly, this configuration accomplished the spatial separation between plasmonic and photonic components at the emission output ports. When the waveguide modes within the CdSe nanowire were excited, these guided modes were reflected at the end-facets of both CdSe and silver nanowires which satisfies the condition of optical cavity. To investigate the lasing behaviors of the combined structure, a 532 nm pulsed laser (5 ns pulse width, 2 kHz repetition rate) was employed to pump the CdSe nanowire by focusing the laser spot on the left segment of the nanowire (Figure 6h). The typical emission spectra from the right end of the silver nanowire were plotted in Figure 6j. Clear light output was observed from the right end-facet of the silver nanowire (see Figure 6l), distinctly indicating the strong photon-plasmon coupling. Multiple lasing modes centered around 723 nm were obtained from both the CdSe and silver nanowires, as shown in Figures 6j,k. The average FSR of the CdSe-Ag hybrid cavity was estimated to be 1.5 nm which matches well with the theoretical calculation by FSR ≈ $\frac{\lambda^{2}}{2}\left(L_{CdSe}n_{g,CdSe}+L_{Ag}n_{g,Ag}\right)$ (λ, *L*_*CdSe*_, *n*_*g, CdSe*_, are the lasing wavelength, length, and group index of CdSe nanowire; *L*_*Ag*_*, n*_*g, Ag*_ are the length and group index of silver nanowire, respectively), verifying that the lasing originates from the hybridization of CdSe and silver nanowires. It is worth noting that the above equation about FSR only holds true for the materials with low dispersion. In addition, almost the same threshold of 60 μJ cm^−2^ for the CdSe and silver nanowires was observed (see inset in Figure 6l). When the pump fluence was further increased to 97 μJ cm^−2^, an extra peak around 745 nm was measured that coincides with the lasing peak of the bare CdSe nanowire (gray line in Figure 6k), suggesting the coexistence of the hybrid cavity mode and single CdSe nanowire mode. Notably, the mode around 745 nm was absent in the emission spectra of the silver nanowire, confirming that the output of silver nanowire stems from the hybrid cavity. Moreover, the lasing threshold of single CdSe nanowire was about 90 μJ cm^−2^ due to the absence of silver nanowire with higher reflection coefficients end-facets. Therefore, the low threshold and different emission spectra of the combined structure became the solid evidences to prove the lasing behavior of the proposed hybrid cavity. To further verify the property of the output from silver nanowire, the polarization-dependent lasing emissions were conducted, as shown in Figure 6m. A polarization ratio of 92% was obtained by comparing the lasing intensities perpendicular to the nanowire axis and parallel polarization, manifesting the feature of the plasmonic mode (Oulton et al., 2009).

##### Multicolor plasmonic lasers

**Figure 7:**
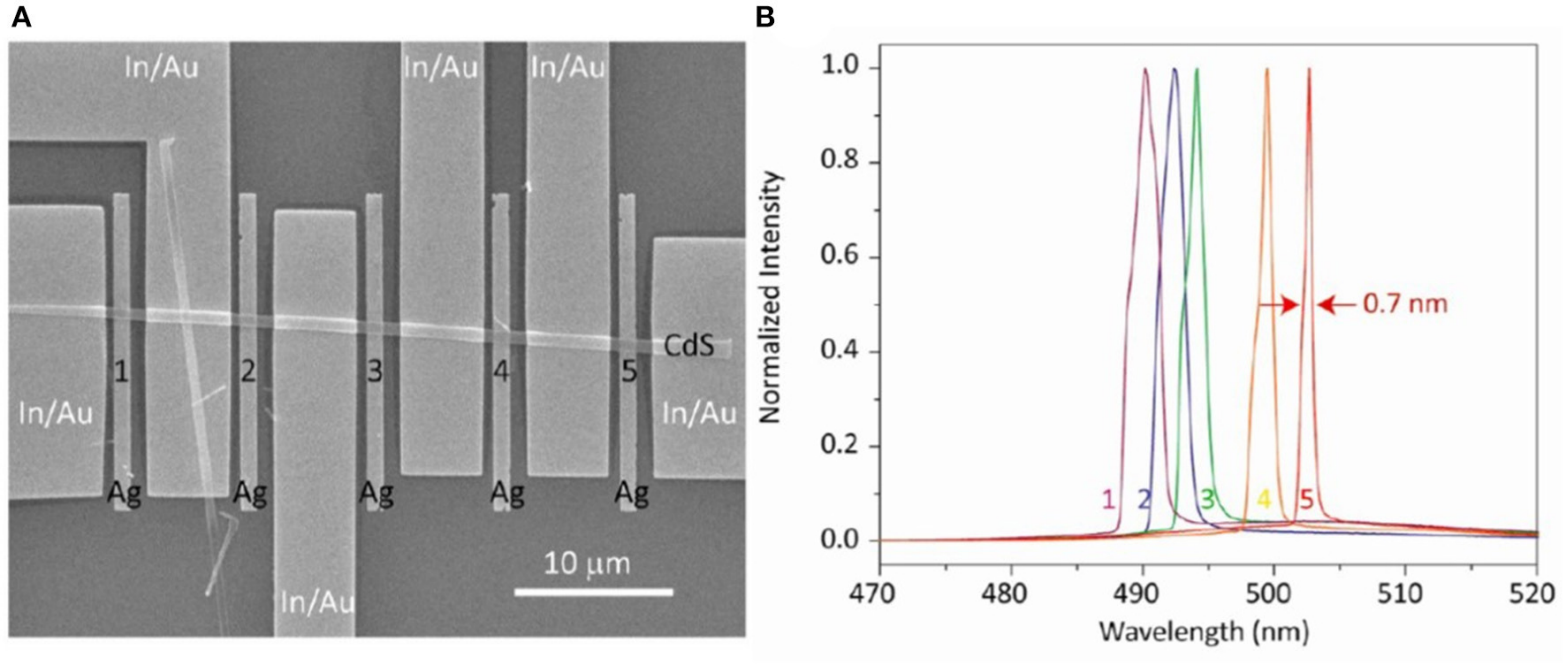
**(A)** SEM image of the multicolor plasmon laser. A single CdS nanowire is divided into five plasmonic cavities with different widths. Six In/Au electrodes are fabricated to modulate the emission of the cavities. **(B)** Spectra of these five plasmonic lasers. **(A,B)** Reproduced with permission. Ma et al. (2012) Copyright 2012, American Chemical Society.

Nowadays, lasers have been widely used for applications including illumination, information, and display (Sirbuly et al., 2005; Neumann et al., 2011; Yang et al., 2011; Dang et al., 2012; Zhang et al., 2015), steadily increasing the need for lasers with high performance and multifunction. Multicolor laser is one of the essential targets to be achieved and significant progress has been made in this area. In 2012, Ma et al. have demonstrated a room-temperature CdS plasmonic laser with multicolor emissions (Ma et al., 2012). The CdS nanobelt was crosswise integrated onto five silver strips with width of 1 μm and thickness of 250 nm separated by a MgF_2_ layer (5 nm). Six In/Au electrodes were fabricated to introduce controllable electrical modulation, as shown in Figure 7A. Five plasmonic cavities have been constructed at the overlap areas of silver and CdS, which were marked as 1–5 in Figure 7A. The cavity sizes were determined by the width of the areas from 1 to 5. The hybrid plasmonic mode could be generated in the overlap area of Ag/MgF_2_/CdS when the device was optically pumped. Clear lasing peaks spanning from 491.2 to 502.7 nm were observed and the wavelengths were decided by the width of the CdS nanobelt (see Figure 7B). Moreover, the authors also provided a solution to tailor the wavelength by applying electric field (4 V). A wavelength shift of ~0.3 nm has been achieved by electric field enhanced density of excited carriers. In this configuration, more than 70% of its radiation could be directed in the embedded semiconductor nanobelt. Such hybrid photonic-plasmonic circuit offers the platform to realize devices with multifunction such as multicolored plasmonic lasers, electrical modulation, and efficient waveguide collection, indicating a significant step toward on-chip integrated optoelectronic circuitry.

**Figure 8:**
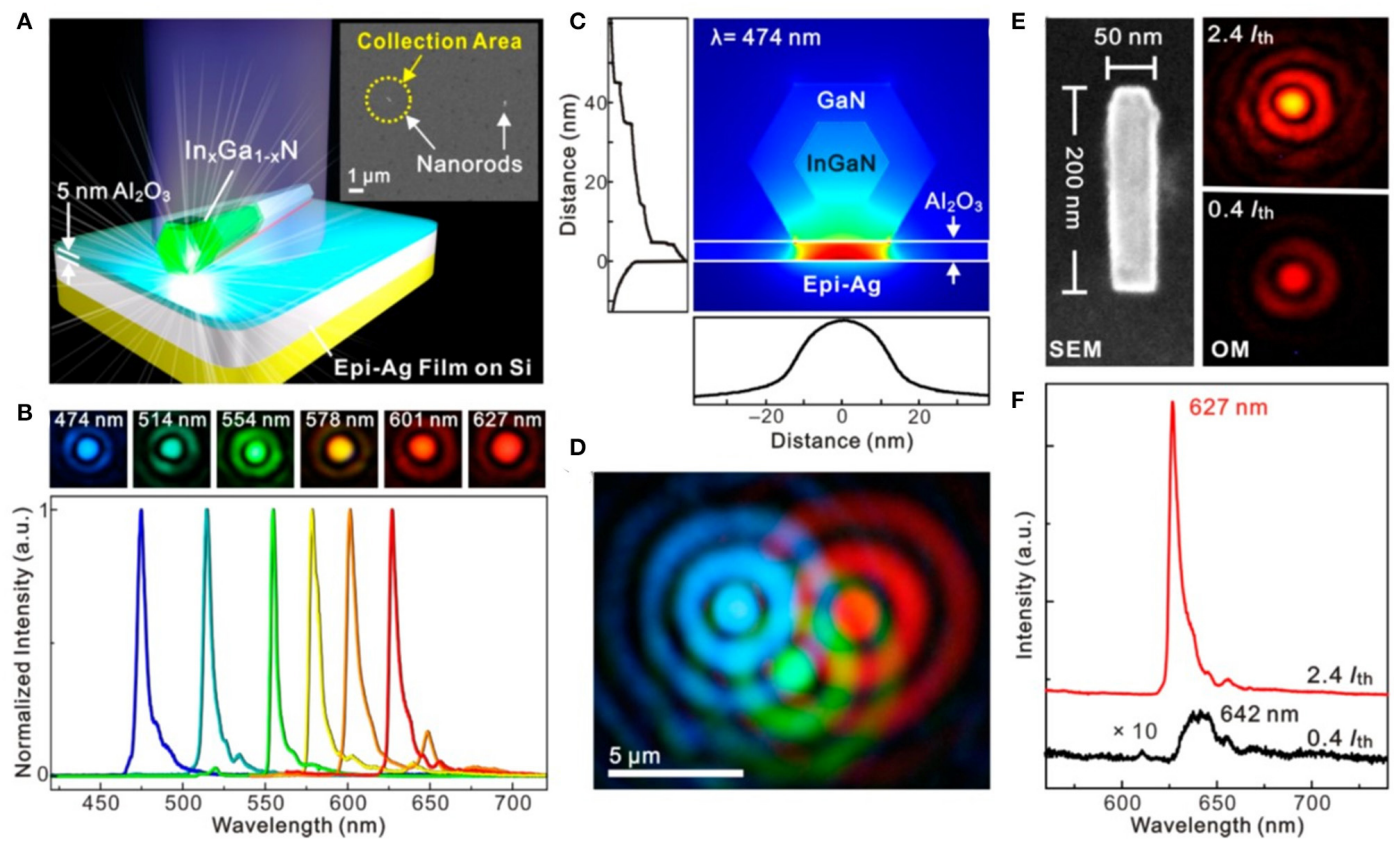
**(A)** Schematic of the plasmonic nanolaser. Inset shows the SEM image of the structure. Scale bar: 1 μm. **(B)** All-color, single-mode lasing emissions with HWFM ~4 nm from single nanorods. **(C)** Simulated mode field distribution of the nanolaser at wavelength of 474 nm. **(D)** Concurrent RGB lasing from three nanorods placed close to each other. Scale bar: 5 μm. **(E)** left panel: SEM image of the device. Optical microscope image below (0.4 *I*_*th*_, upper right panel) and above (2.4 *I*_*th*_, lower right panel) lasing threshold. **(F)** Spectra of the nanolaser. Red line: spontaneous emission (642 nm). Black line: lasing (627 nm). **(A–F)** Reproduced with permission. Lu et al. (2014) Copyright 2014, American Chemical Society.

In addition to the multicolored plasmonic lasers by tuning cavity size, another strategy to achieve all-color plasmonic nanolasers has been proposed by Lu et al. (2014), as shown in Figure 8A. The indium gallium nitride (In_*x*_Ga_1−x_N, 0 ≤ *x* ≤ 1) compound semiconductor alloy system has been utilized to realize plasmonic lasers from blue (474 nm) to red (627 nm). The direct band gap of such material can be tuned by different indium contents in the nanorods. By multiple drop casting, they fabricated the In_*x*_Ga_1−x_N/Al_2_O_3_/Ag plasmonic nanolasers with different emission colors. As confirmed by Figure 8B, single-mode lasing emissions in the full visible region (blue, cyan, green, yellow, orange, and red) have been observed. The Airy disk diffraction patterns in the optical images in Figure 8B clearly prove the subwavelength scale of the physical sizes of these devices. The numerical calculation revealed the strong light confinement in the Al_2_O_3_ insulator layer (see Figure 8C). To demonstrate the potential application in display, three diluted suspension solutions of nanorods with different indium contents were sequentially applied to deposit RGB nanorods within close proximity of each other, as shown in Figure 8D. From the SEM image shown in Figure 8E, the nanorod length and diameter were determined to be ~200 and ~50, unambiguously verifying the subwavelength footprint of the device. Optical microscopy images and the emission spectra (see lower panel in Figure 8E) indicates that the frequency pulling effect (Stockman, 2010; Lu et al., 2012) has occurred from spontaneous (642 nm) emission to lasing (627 nm). In addition, lasing with ultralow thresholds have been achieved by optimizing the composing materials in In_*x*_Ga_1−x_N/Al_2_O_3_/Ag configuration. These results open up a route to a wide range of potential applications in ultrafast information processing, nanoscopy, nanolithography, biomedicine, and sensing.

#### Single-mode lasers

**Figure 9:**
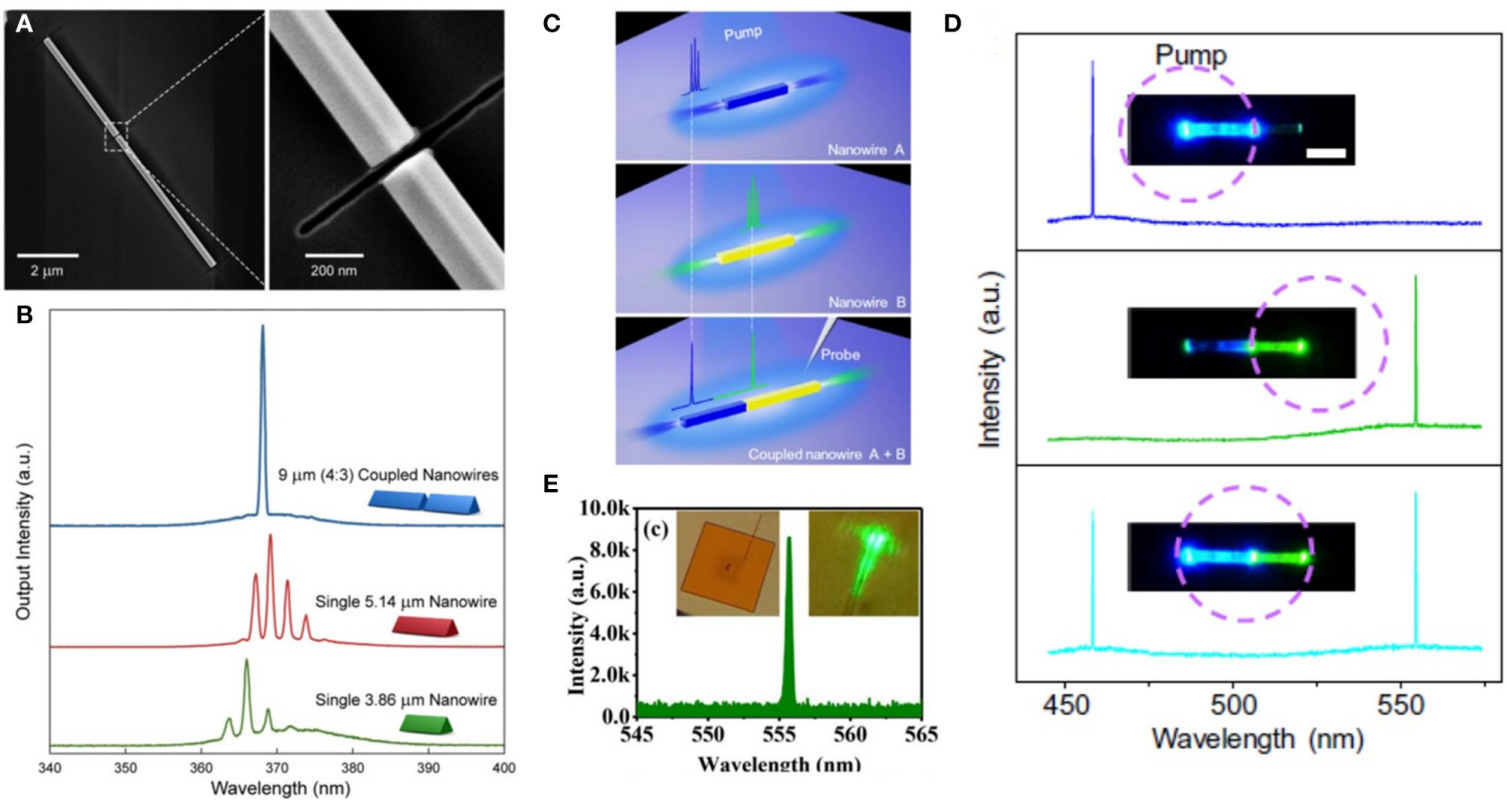
**(A)** SEM images of the axially coupled GaN nanowires on silicon substrate. Scale bar: 2 μm (left panel) and 200 nm (right panel). **(B)** Lasing spectra from single GaN nanowire with length 3.86 μm (green line), 5.14 μm (red line), and coupled nanowires (blue line). **(C)** Sketch of the multimode lasing from separate A and B nanowires (top and middle) and dual-color single-mode lasing from axially coupled A + B nanowire cavities (bottom). **(D)** Lasing emission spectra of the axially coupled heterogeneous nanowire cavities under excitation at different positions. Scale bar: 10 μm. **(E)** Spectrum of the single-mode lasing emission at a pump density of 12 μJ cm^−2^. Inset: optical microscope image and fluorescent image of the nanowire above the lasing threshold. **(A,B)** Reproduced with permission. Gao et al. (2013) Copyright 2012, National Academy of Sciences. **(C,D)** Reproduced with permission. Zhang C. et al. (2017) Copyright 2017, American Association for the Advancement of Science. **(E)** Reproduced with permission. Wang et al. (2016) Copyright 2016, Optical Society of America.

Single-mode lasing is highly desirable for applications in sensing, optical communications, spectroscopy, and interferometry. Semiconductor nanowire lasers featured with optical gain usually exhibit multiple lasing modes because of the relatively long cavity length. Reducing nanowire length to expand the FSR means that only one longitudinal mode exists in the gain window of the nanowire (Li et al., 2012). However, the shortened cavity length would increase the lasing thresholds of the nanowire lasers due to the reduction of round-trip gain. In 2013, Gao et al. developed a strategy to realize mode selection by cleaving a GaN nanowire at a determined point (Gao et al., 2013). As shown in Figure 9A, a GaN nanowire on a Si substrate was cleaved by focused ion beam (FIB) milling. To prevent the degradation of the luminescent properties of the nanowires, a metallic layer was fabricated before ion beam milling. This technique provides the precise control of the cavity length and intercavity gap. The intentionally created gap is around 40 nm, ensuring the intercavity coupling between two disconnected nanowires. In addition, the well-cleaved end-facets also contribute to the low scattering loss of the optical cavities. When the two nanowires (with lengths of 3.86 and 5.14 μm, respectively) were individually pumped, multiple F-P lasing modes have been observed (Figure 9B, green and red lines). Interestingly, when both of the two nanowires were excited, single-mode lasing was generated from the coupled nanowire cavity (blue line in Figure 9B). This study provides an effective way to improve the emission quality by increasing the optical gain in a tailored geometry. In 2016, Wang et al. demonstrated another mode selection mechanism to realize a single-mode laser (Wang et al., 2016). A single-crystalline perovskite nanowire was transferred onto a perovskite microplate by micro-manipulation via a home-made fiber probe (Figure 9E). Due to the identical refractive index of the nanowire and microplate, the quality (*Q*) factors of the F-P modes were spoiled. Then the natural F-P modes along the longitudinal direction of the nanowire could be significantly suppressed, because of the strong leakage to the perovskite microplate (Gu et al., 2015). Consequently, only the mode in the transverse plane of the nanowire survives and could lase. As demonstrated in Figure 9E, single-mode lasing was obtained when the sample was optically excited by a femtosecond pulsed laser. The local bright emission spot instead of two emission spots of F-P lasers has been clearly captured by optical microscopy (inset in Figure 9E).

Shortly after, Zhang et al. reported another strategy to enable dual-color single-mode lasing, as schematically illustrated in Figure 9C (Zhang C. et al., 2017). An axially coupled nanowire cavity was constructed by two nanowires self-assembled from distinct organic materials. With the assistance of a micromanipulator, these two resonators were integrated to achieve a significant mode selection effect. Lasing actions with multiple peaks at different wavelengths could be readily observed from a single nanowire cavity, as shown in the top and middle panels of Figure 9D. After axially aligning the two nanowires, each nanowire served as a spectral filter to select the mode in the coupled cavity and the single-mode emission could be expected. The nanowire on the left side was solely excited and modulated by the nanowire on the right side. Single-mode lasing (458 nm) was clearly established at this time, as shown in the top panel in Figure 9D. When the nanowire on the right side was pumped, the roles of two nanowires reversed and contributed to the single-mode lasing at 554.8 nm. Moreover, this mutual mode selection effect in the axially coupled heterogeneous nanowire cavities would enable the output of the dual-wavelength single-mode laser when the two coupled nanowires were simultaneously optically excited (see the bottom panel in Figure 9D). The proposed mechanism not only allows for achieving multicolor single-mode lasing but also permits the controllable outcoupling of the modulated lasers at nanoscale, providing new ideas for the construction of photonic elements with desired functionalities.

**Figure 10:**
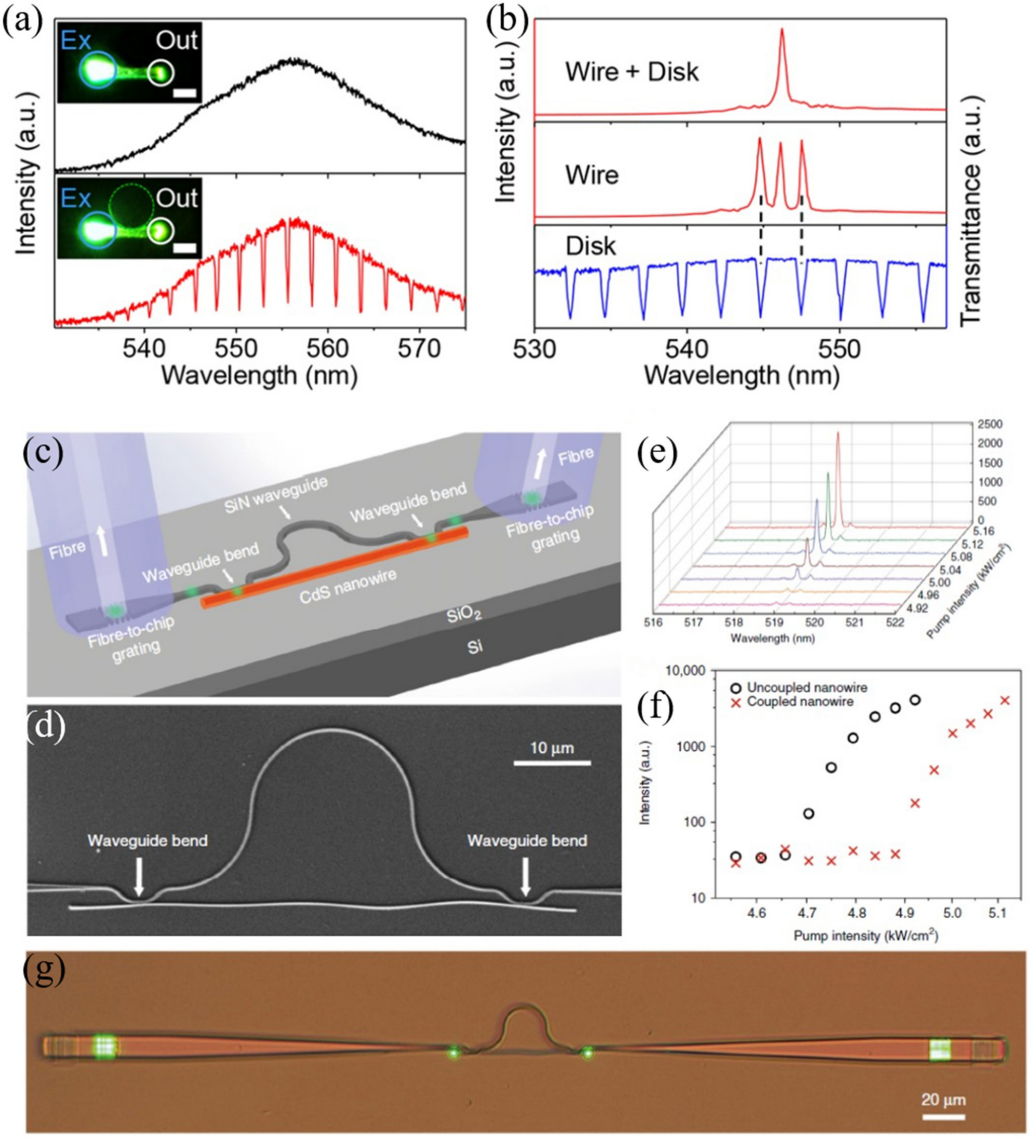
**(a)** Original and modulated PL spectra from perovskite nanowire and microdisk coupled nanowire. Insets show the microscope images. **(b)** Schematic of the mode selection effect in the nanowire-microdisk system. **(c)** Schematic diagram and **(d)** SEM image of the hybrid MZI structure. **(e)** Lasing spectra obtained at different pumping intensities above the threshold. **(f)** Dependence of the lasing output on the pumping intensity of the excited CdS nanowire for the coupled (red) and uncoupled (black) cases. **(g)** Optical image of the hybrid MZI structure under excitation. **(a,b)** Reproduced with permission. Zhao et al. (2018) Copyright 2018, American Chemical Society. **(c–g)** Reproduced with permission. Bao et al. (2020) Copyright 2020, Nature Publishing Group.

Based on the similar mode selection mechanism, Zhao et al. demonstrated switchable single-mode microlasers from perovskite nanowire in 2018 (Zhao et al., 2018). A vapor-responsive organic microdisk tangentially coupled with a perovskite nanowire. The nanowire serves as both gain medium and F-P cavity, while the coupled organic microdisk functions as a spectral filter to the lasing modes of the perovskite. As a result, single-mode lasing could be achieved in such a combined nanowire-microdisk system, as illustrated in Figure 10a. The single nanowire shows broad PL spectrum without the coupled microdisk. However, the PL spectrum was clearly modulated and dips were observed when the microdisk tangentially coupled with the nanowire. Therefore, some lasing modes of the nanowire would be suppressed by the spectra dips at the same wavelengths formed via microdisk modulation, leading a single-mode lasing, as shown in Figure 10b. In addition, the microdisk shows inherent sensitivity to acetone gas and exhibits changes of effective radius with the acetone concentration. In this sense, the transmittance spectra of the microdisk could be varied when the gas was introduced. Consequently, the survived mode of the nanowire could be switched by tuning the gas concentration, confirming the potential application of the sensing function.

In addition to the above mechanisms to achieve single-mode lasing, Bao et al. proposed a mode selection scheme using a Mach-Zehnder interferometer (MZI) structure (Bao et al., 2020), as shown in Figure 10c. A CdS nanowire is used as gain medium and is evanescently coupled with an Ω-shaped silicon nitride (SiN) to form the hybrid MZI structure, as the SEM image shown in Figure 10d. The bent areas of the SiN waveguide ensure the high coupling efficiency (Chen et al., 2017). When both sides of the nanowire were fully coupled with the SiN waveguide, the established MZI structure selects only one dominant lasing mode by suppressing all other modes of the CdS nanowire. Under optical pumping, bright green light emissions have been observed from the two end-facets of nanowire and two grating couplers (see Figure 10e). Moreover, a clear single-mode feature was proved by the measured spectra, as shown in Figure 10f. The maximum value of the side-mode suppression ratio (13 dB) could be reached at the pumping density of 5.2 kW cm^−2^. The “S-like” curve in Figure 10g confirms the lasing action of the on-chip nanowire laser. Compared with previously reported nanowire lasers, this work achieves much higher efficiency and can be operated in the single-mode regime with a small footprint and high flexibility. Furthermore, such an on-chip integration scheme can be extended to other semiconductor nanowire materials and readily realize integrated laser from UV to near IR regions.

#### Wavelength-Tunable Lasers

**Figure 11:**
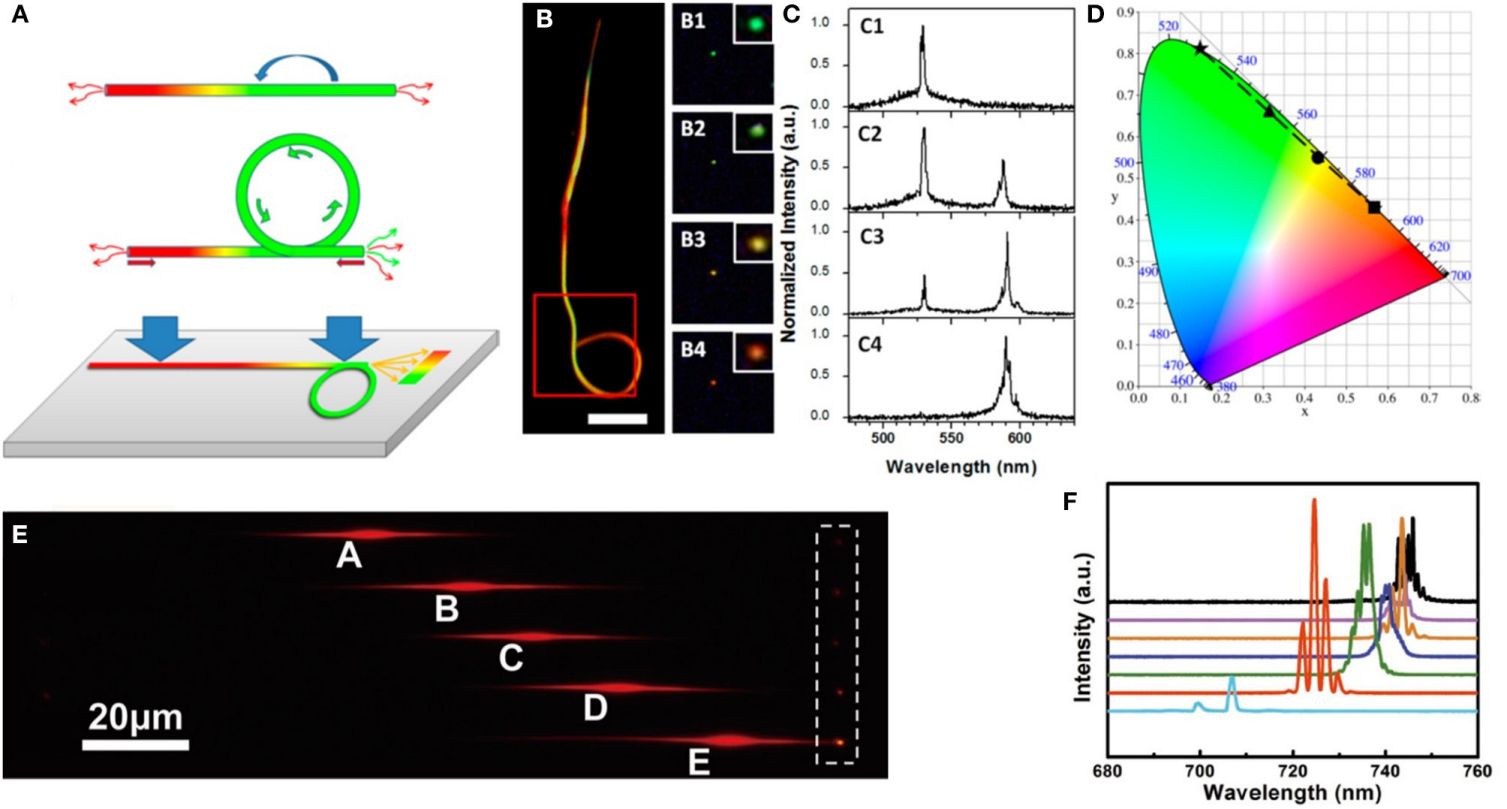
**(A)** Up: Sketch of a straight CdSSe alloy nanowire. Middle: Sketch of the looped nanowire. Bottom: Design of the color-tunable laser using this looped nanowire by changing the relative strengths of pumping of the two segments. **(B)** Dark-field image of the looped nanowire, Scale bar: 10 μm. (B1-B4) Real color images of the lasing under different pumping for the two cavities. **(C)** Normalized lasing spectra under different controlled pumping intensities. **(D)** The calculated colors from the spectra in C1-C4 plotted on CIE1931 color space. **(E)** PL microscope images of a single 150-μm-long, 402-nm-diameter CdSe nanowire excited at different positions along its length. **(F)** Laser spectra with different nanowire lengths under the same pump beam size and intensity. Nanowire lengths for the spectra from left to right in the figure are 8, 20, 48, 102, 147, 179, and 289 μm, respectively. **(A–D)** Reproduced with permission. Liu N. et al. (2013) Copyright 2013, American Chemical Society. **(E,F)** Reproduced with permission. Li Y. et al. (2013) Copyright 2013, Wiley-VCH.

The nanoscale size of nanowire offers the opportunity to realize devices with ultrasmall footprints and mode volumes. However, such properties aggrandize the difficulty to modify the optical properties such as wavelength of the nanowire. A number of approaches have been proposed to achieve wavelength tunability by modifying the dielectric environment via excitation intensity (Johnson et al., 2003), cavity size (Li et al., 2013), or substrate properties (Liu X. et al., 2013). In addition, tailoring the material composition and bandgap is another route to modify the emission color. In the past two decades, an expanse of semiconductor nanowire lasers with tunable wavelength have been demonstrated, for instance, Zn_*x*_CdS_1−x_ (Liu et al., 2005), Cd_*x*_SSe_1−x_ (Pan et al., 2005, 2006, 2009b; Liu et al., 2007; Zapien et al., 2007), In_*x*_Ga_1−x_N (Kuykendall et al., 2007), and metal halide perovskite (Xing et al., 2015; Zhu et al., 2015). In 2013, Liu et al. reported a novel design by engineering the material composition and cavity shape to realize lasing with a wide tuning range (Liu et al., 2013). On one hand, the composition along the CdSe alloy nanowire axis was purposely engineered through nanoscale manipulation. The sulfur composition *x* of CdS_*x*_Se_1−x_ can be tuned continuously from 1 to 0, enabling a wide and continuous tuning range from green to red. On the other hand, one end of the nanowire was looped to create two weakly coupled optical cavities, as shown in Figures 11A,B. Thus, the absorption of the short wavelength in the narrow-gap section can be decreased, resulting in the lasing actions of two cavities simultaneously. Two excitation beams with different intensities were employed to separately pump the CdSe-rich straight part and CdS-rich loop, as shown in Figure 11B. By adjusting the intensities of two beams, the output color at the junction area (red box in Figure 11B) can be tuned as green, yellow-green, yellow, and orange, as the optical images shown from B1 to B4 in Figure 11B. The corresponding lasing spectra (C1-C4 in Figure 11C) of B1-B4 uncovers that the essence of tunable color originates the mixture of orange and green light with different weights by varying the pumping power difference of two beams. Additionally, the obtained colors from the nanowire laser match well with the CIE1931 color space and any color on the dashed line (Figure 11D) is available by precisely controlling the intensity ratio of two excitation beams.

**Figure 12:**
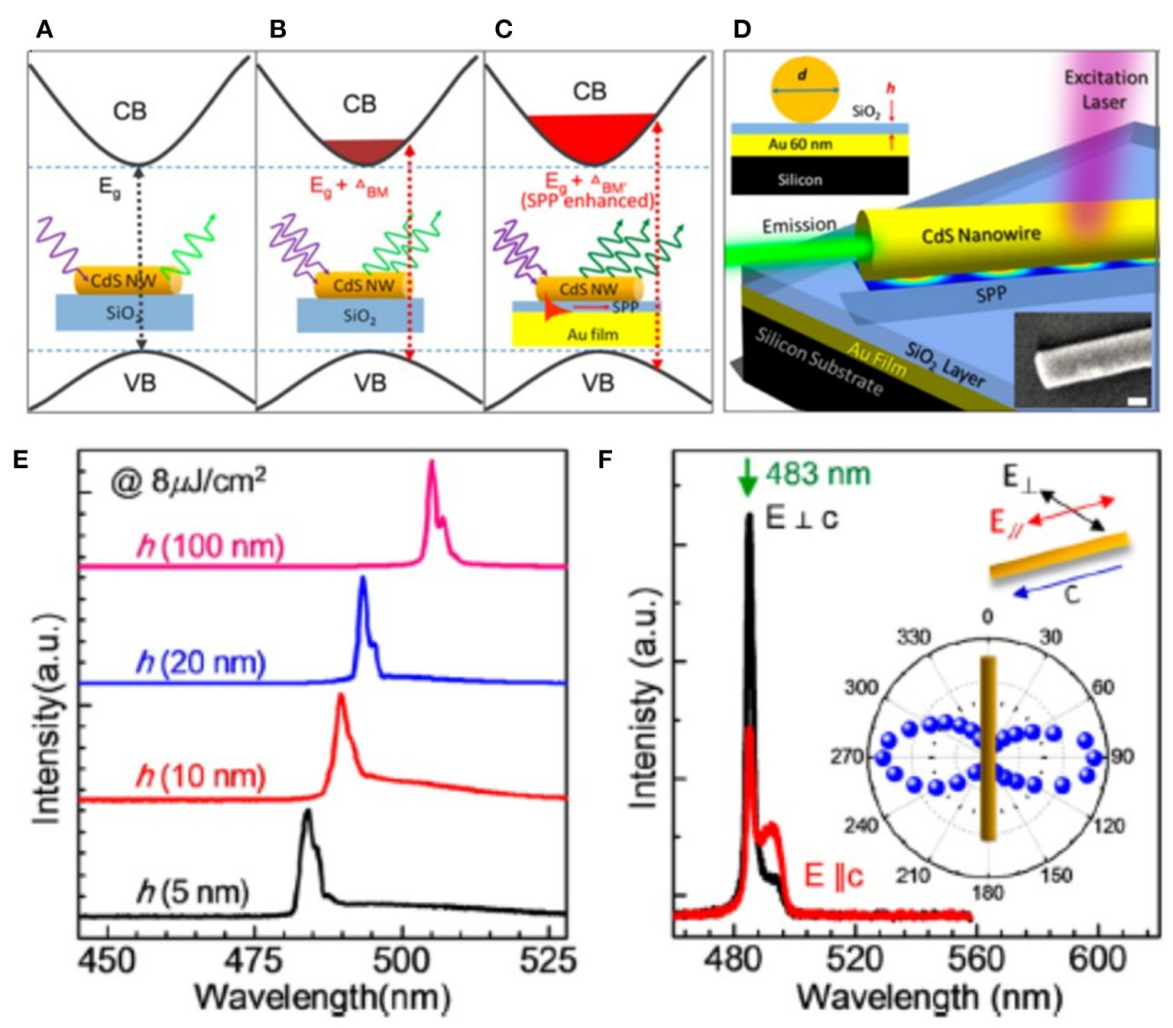
An illustration of the various effects on the band gap energy of non-doped semiconductor nanowires: **(A)** without the BM effect (under low pump fluence), **(B)** with the BM effect (under high pump fluence), and **(C)** the SPP enhanced BM effect. **(D)** A schematic of the nanowire laser devices. Inset shows the SEM image of a single CdS nanowire on the gold film. Scale bar: 100 nm. **(E)** The lasing peaks of four devices under the same pump fluence of 8 μJ cm^−2^. **(F)** Polarization-dependent lasing properties of the device. Inset is the far-field distribution of the lasing emission at ~483 nm. **(A–F)** Reproduced with permission. Liu X. et al. (2013) Copyright 2013, American Chemical Society.

Different from engineering the axial composition of alloy nanowire, Li et al. demonstrated a wide-wavelength tunable laser from a single nanowire based on absorption-emission-absorption (AEA) (Li et al., 2013). As shown in Figure 11E, a single 150-μm-long, 402-nm-diameter CdSe nanowire was excited at different positions along the axis direction (as marked by A-E in Figure 11E). Then the PL were collected from the right end of the nanowire, as the dashed box shown in Figure 11E. Because the self-absorption of the higher energy photons and re-emission of low energy photons are related to the propagation distance, clear PL red-shifts from 714 to 740 nm (see the spectra plotted in Figure 11F) have been observed by changing the distance between pumping point and collection point from 5 to 130 μm. Such a scheme provides a flexible way to tailor lasing wavelength and is promising in applications ranging from communication to detection (Hinkley and Kelley, 1971; Chu et al., 2009). In the same year, Liu et al. demonstrated a wavelength tunable laser based on the notion of the Burstein-Moss (BM) effect (Liu X. et al., 2013). Under strong optical pumping, the Fermi energy level in the conduction band may arise due to the state-filling close to the bottom of the conduction band, consequently leading to the blue shift of the optical gap (Burstein, 1954), as illustrated in Figures 12A–C. The strength of the BM shift is proportional to $n_{e}^{2/3}$, where *n*_*e*_ is the electron carrier concentration in the conduction band which is proportional to the pump fluence and inversely proportional to the nanowire diameter. In this sense, the wavelength of nanowires could be manipulated by varying the pumping intensity or the nanowire size (Sun et al., 2013). As demonstrated in Figure 12D, Liu et al. fabricated the hybrid plasmonic devices with different thicknesses of the insulator layer (5, 10, 20, 100 nm). To avoid the influence of nanowire size, a series of CdS nanowires with approximately the same length (i.e., ~10 ± 0.5 μm) and diameter (i.e., ~220 ± 10 nm) were selected. When decreasing the SiO_2_ insulator gap from 100 to 5 nm, the local carrier concentrations could be dramatically enhanced and this subsequently improved the BM effect. Thus, given an excitation above the lasing threshold (8 μJ cm^−2^), a considerable tuning range of >20 nm has been observed, as illustrated in Figure 12E. Notably, the lasing modes were confirmed to be photonic rather than plasmonic. They collected the polarization-dependent emission spectra, as summarized in Figure 12F. It is seen that the emission polarization for an insulator gap of 5 nm was perpendicular to the axis of the nanowire, which matches well with the characterization of transverse electric mode, i.e., photonic mode (Oulton et al., 2009). The strong energy transfer from SPPs to CdS excitons highly enhances the BM effect and provides a valid way to manipulate the light-mater interactions at nanoscale.

### WGM Lasers

**Figure 13:**
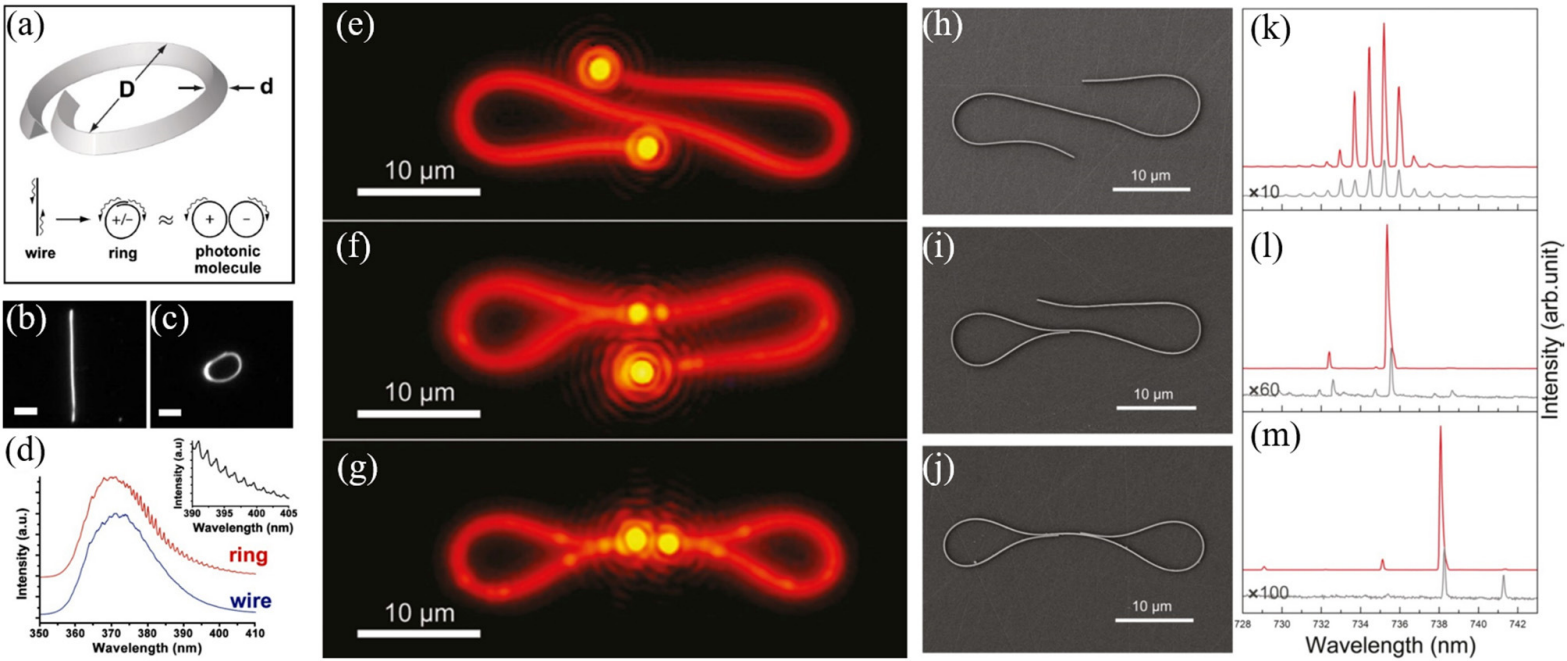
**(a)** Schematic of the ring cavity based on GaN nanowire. The GaN nanowire before **(b)** and after **(c)** physical manipulation. Scale bar: 5 μm. **(d)** PL spectra of the nanowire and ring. Inset shows the high-resolution spectrum of the ring. PL microscope images of lasing cavities of single-nanowire structures **(e)** without loop mirror, **(f)** with one loop mirror, and **(g)** with double loop mirrors. **(h–j)** are their corresponding SEM images. Scale bar: 10 μm. **(k–m)** are their corresponding lasing spectra. **(a–d)** Reproduced with permission. Pauzauskie et al. (2006) Copyright 2006, The American Physical Society. **(e–m)** Reproduced with permission. Xiao et al. (2011) Copyright 2011, American Chemical Society.

Compared with WGMs, the nanowire F-P cavity shows a relatively low Q-factor, because the low reflectivity from the end facets of the semiconductor nanowire create a giant light leakage into the free space and a high lasing threshold. Due to the flexibility of manipulating the nanowire into on-demand shapes and tailoring nanowire into a closed loop, nanowires have been an excellent platform to obtain WGMs and have also attracted tremendous attentions in the recent years. Usually, a circular optical cavity can be formed by connecting the two ends of the nanowire. The evanescent coupling between two ends ensures sufficient energy exchange (Law et al., 2004; Gu et al., 2015) and positive feedback. As shown in Figures 13a–c, a ring structure was constructed by overlapping the two ends of the GaN nanowire with the micromanipulator (Pauzauskie et al., 2006). The periodic mode peaks have been observed from this ring cavity (Figure 13d) and the measured mode spacings match well with calculated value by $\Delta \lambda =\frac{\lambda_{0}^{2}}{2\pi R\left(n-\lambda_{0}\frac{dn}{d\lambda }\right)}$, clearly confirming the formation of WGMs in this structure. In 2009, the similar WGMs lasing from CdS nanowire has also been reported by Ma et al. (2009). Furthermore, they constructed a ring-F-P coupled structure to guide the output and modulate the WGMs of the ring cavity. Later, Ma et al. demonstrated a pigtail structure with high collection efficiency by coupling a tapered nanofiber (Pigtailed, 2010). In 2011, a single-nanowire single-mode laser was demonstrated by Xiao et al. (2011) By folding the CdS nanowire into loops (Figures 13e–j), mode selection is realized by the Vernier effect of the coupled cavities and a single-mode emission of around 738 nm was obtained. The ∞-shaped cavities enlarged the FSR to 3 nm and suppressed the lasing modes of the two individual cavities. Thus, only one dominant mode was selected within the lasing range. As confirmed in Figures 13k–m, the lasing spectra varied with the change of the cavity shapes. Multiple modes with FSR of ~0.75 nm can be observed for the case in Figure 13h, corresponding to the calculated value by FSR$\approx \frac{\lambda^{2}}{2Ln_{g}}$, where *L* = 75 μm is the nanowire length and *n*_*g*_ ≈ 4.8 is the group index of the CdSe nanowire. Once the loop mirrors have been established, single mode lasing with a side mode suppression ration of up to 20 dB was achieved, as the spectra plotted in Figure 13m.

**Figure 14:**
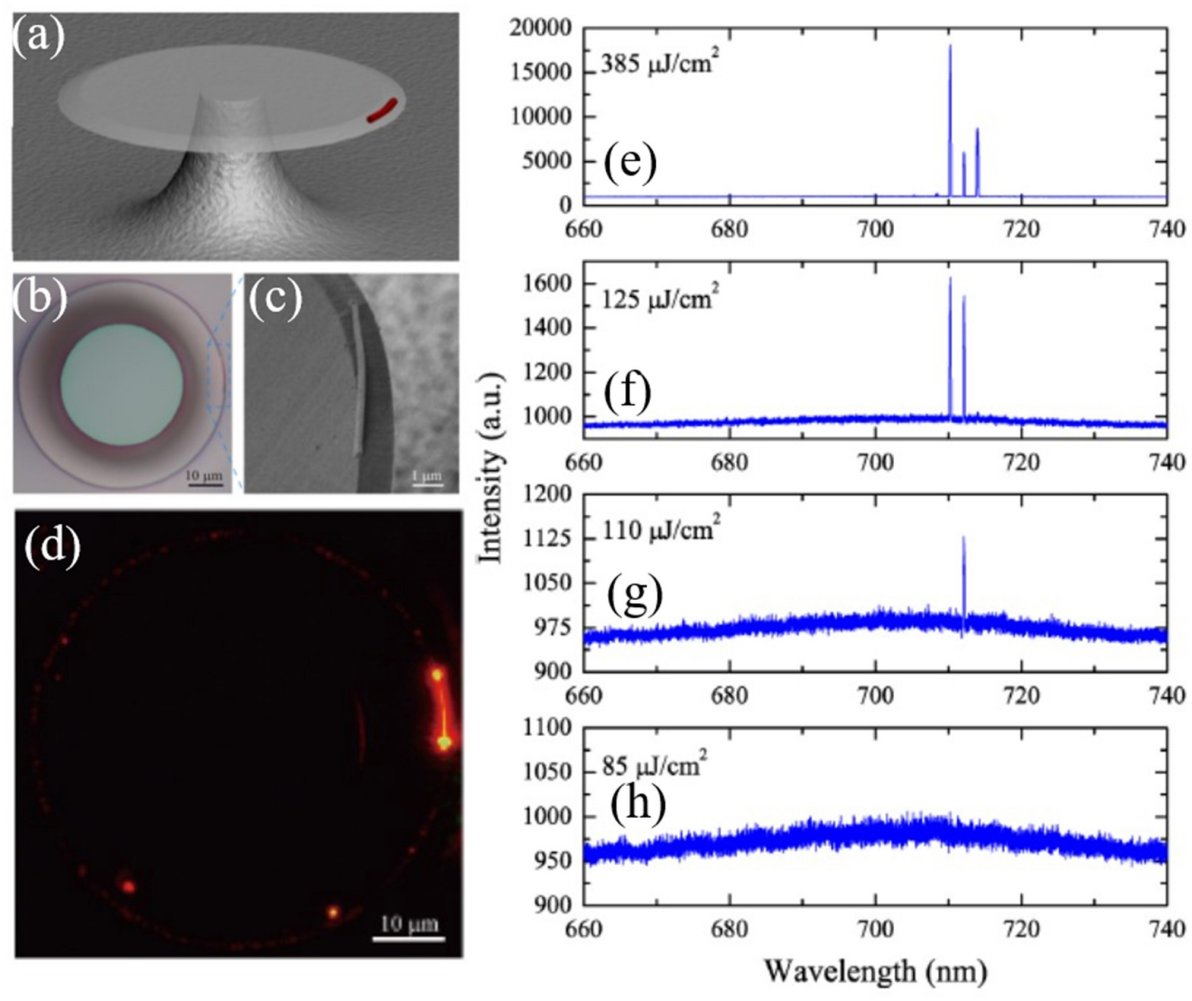
**(a)** Schematic of the hybrid structure consisting of a nanowire and microdisk. **(b)** Optical microscope image of the hybrid structure. **(c)** SEM image of the hybrid structure. **(d)** PL image of the hybrid structure above lasing threshold. **(e–h)** Measured spectra of the hybrid cavity under different pumping powers. **(a–h)** Reproduced with permission. Wang et al. (2012) Copyright 2012, Optical Society of America.

In addition to tailoring the nanowire into circular shapes, another strategy of transferring semiconductor nanowire onto circular cavity has been developed. In 2012, Wang et al. proposed a hybrid structure consisting of a single CdSe nanowire and a silica microdisk cavity (Wang et al., 2012), as shown in Figures 14a–c. Such a combined system simultaneously possesses the large gain of nanowire and high-*Q* of the microdisk. A *Q*-factor higher than 10^5^ at 1.5 μm was achieved, enabling a low threshold on-chip lasing at room temperature. The SEM image of the hybrid structure in Figure 14b shows that the nanowire lay on edge of the microdisk which assured the large overlap between the gain in nanowire and the WGMs in microdisk. When the hybrid cavity was optically pumped, the WGMs with the highest *Q*-factor lased first under excitation density of 110 μJ cm^−2^, as shown in Figure 14e. Given a higher pumping power, multiple lasing peaks with FSR of ≈8.9 nm and linewidth of ≈0.18 nm have been observed. Clear light scattering can be seen at the two end-facets of the nanowire and the rim of the microdisk (Figure 14d). Figures 14f–h show the spectra evolution of the emitted light from the microdisk-nanowire system.

## Applications

**Figure 15:**
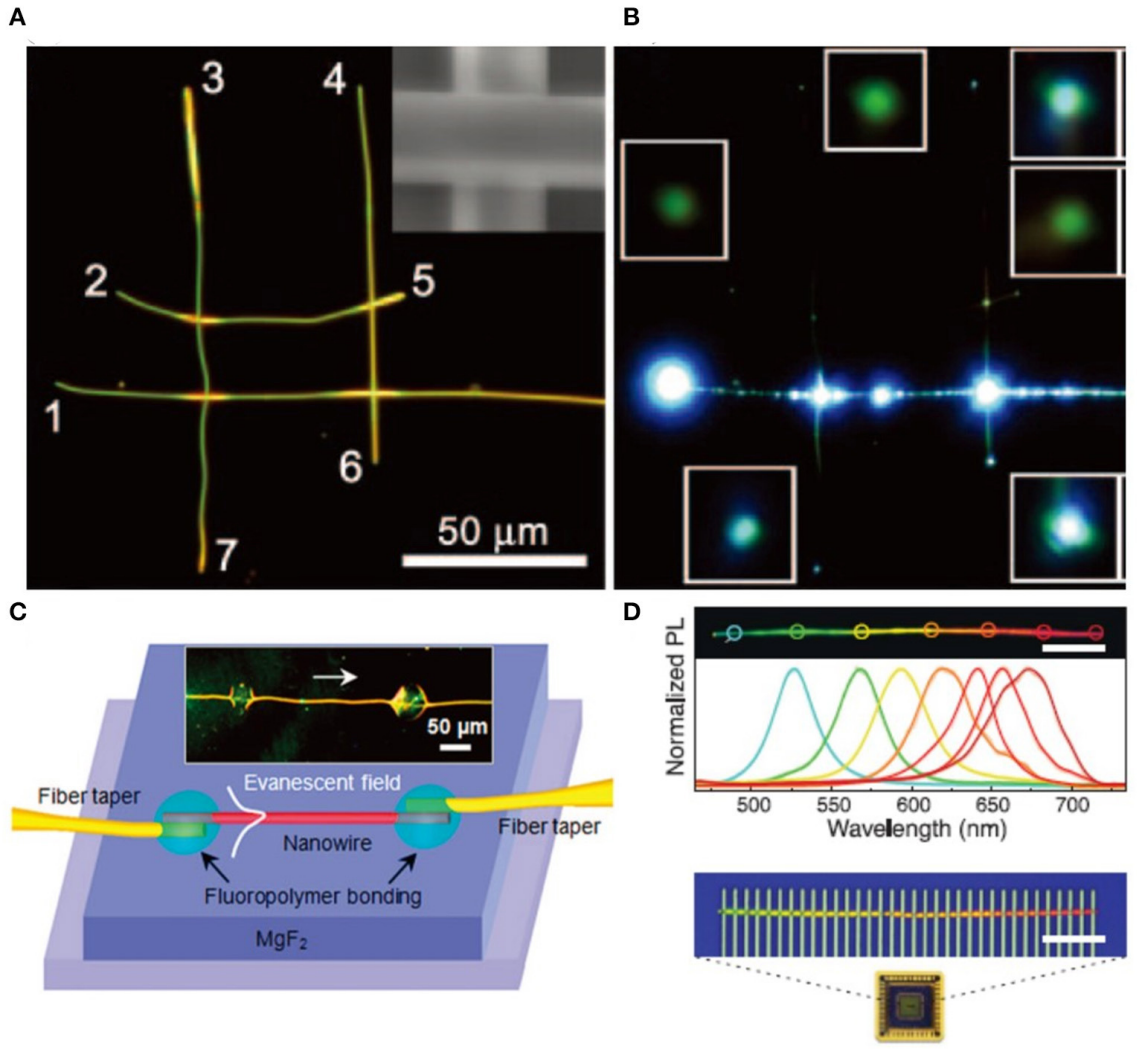
**(A)** Dark-field image of the four-ribbon structure. Light is inputted from the right long side and the output channels are labeled as 1–7. Scale bar: 50 μm. **(B)** PL image as the input channel is pumped at 325 nm. **(C)** Schematic illustration of the polymer nanowire sensor. Scale bar: 50 μm. **(D)** Real-color PL image of a typical compositionally graded CdS_*x*_Se_1−x_ nanowire and corresponding spectra collected from marked representative regions (spot size ~5 μm). Scale bar: 20 μm. **(A,B)** Reproduced with permission. Sirbuly et al. (2005) Copyright 2005, National Academy of Sciences. **(C)** Reproduced with permission. Gu et al. (2008) Copyright 2008, American Chemical Society. **(D)** Reproduced with permission. Yang Z. et al. (2019) Copyright 2020, American Association for the Advancement of Science.

Up to now, the nanowires have been thoroughly investigated for their materials, structures, and physics, boosting the rapid development of nanowire-based devices. Also, the significant progress in synthesis methods and fabrication techniques offers a valued opportunity to manipulate light at nanometer scale, which significantly promotes the research on nanowire applications including (but not limited to) photonic networks (Sirbuly et al., 2005; Guo et al., 2009; Gu et al., 2016), sensing (Meng et al., 2011), optical switching (Zhang N. et al., 2019), and display (Yang et al., 2011). The length, flexibility, and strength of semiconductor nanowires make them easier than other nanostructures to build novel and versatile photonic circuitry. As confirmed in Figure 15A, Sirbuly et al. explored the possible application of SnO_2_ in optical routing and networks (Sirbuly et al., 2005). A rectangular grid (46 μm long and ≈ 25 μm wide) was constructed by four nanoribbons. Seven output channels (marked as 1–7 in Figure 15A) could be monitored when the long input channel was directly excited. Different intensity distribution of 1 >> 6 > 4 ≈ 7 > 3 > 5 > 2 has been obtained after considering the trajectory of the incoming light and the intensity of scattering at the four ribbon-ribbon junctions (as shown in Figure 15B). In 2008, Gu et al. reported the highly versatile nanosensors using polymer single nanowires (Gu et al., 2008). As shown in Figure 15C, the subwavelength diameter of the nanowire provides adequate evanescent coupling between the nanowire and the surrounding medium. When the environment humidity changed, the corresponding refractive index difference between the nanowire and environment varied, which modified the leakage, consequently modifying the transmission spectra of the nanowire. The small footprint, fast response, and high sensitivity of the demonstrated nanosensors open up vast opportunities for fast detection in physical, chemical, and biological applications. Recently, Yang et al. reported an ultracompact microspectrometer based on a single compositionally engineered nanowire (Yang Z. et al., 2019), as shown in Figure 15D. The CdS_*x*_Se_1−x_ nanowires with a continuous gradient of bandgaps spanning from 1.74 to 2.42 eV have been synthesized and transferred onto Si/SiO_2_ substrate. The unknown incident light *F*(λ) can be reconstructed by solving the a system of linear equations after measuring the generated photocurrent data. The demonstrated ultracompact functional device offers a significant step forward for the customized design of ultraminiaturized systems.

## Outlook

Research conducted over the past 20 years has contributed greatly to the flourishment of semiconductor nanowires, including physics, synthesis, and applications. In particular, great progress has been made by expanding the range of available materials and cavity structures, achieving greater control over the alloy composition and lasing mode, and reducing lasing thresholds. However, many fundamental issues and questions still remain. (1) The light collection efficiency of the nanowire lasers should be improved to a higher level. Though the end-facet reflections of nanowire are sufficient to form an optical cavity that satisfies energy recycling, the considerable scattering into free space at the facet-air interfaces is a critical leaky channel which causes a huge waste of emissions. (2) Further study is needed to improve the *Q*-factor of the nanowire cavity. It is worth mentioning that most works to improve the *Q*-factor or reduce the lasing threshold of nanowires are demonstrated by ameliorating structural and material properties. Cavity design using other concepts such as avoided resonance crossing (Song and Cao, 2010) can be a possible approach to acquire mode with a high Q factor in arrayed nanowire. (3) More must be done on electric injection of semiconductor nanowire lasers to achieve commercial applications. Electrical injection is almost a necessity for all semiconductor laser applications, especially for integrated photonics. The rapid development of nanowire technologies has provided numerous exciting opportunities for electronic and photonic applications. One of the utmost important goals for developing nanowire is to construct ultracompact, small footprint devices. (4) Significant efforts are still required to fabricate and operate nanowire lasers with high reproducibility and stability, especially on the level of individual nanowires. Overall, considering such remarkable progress in nanowire photonics, there is plenty of room in either nanowire integrated circuitry or individual nanowire devices. Challenges and opportunities coexist in the future research on semiconductor nanowires.

## Author Contributions

ZG, SX, and QS discussed the results and wrote the paper. All authors contributed to the article and approved the submitted version.

## Conflict of Interest

The authors declare that the research was conducted in the absence of any commercial or financial relationships that could be construed as a potential conflict of interest.

## References

[B1] Ainsworth C. A. Derby B. Sampson W. W. (2018). Interdependence of resistance and optical transmission in conductive nanowire networks. Adv. Theory Simul. 1:1700011 10.1002/adts.201700011

[B2] Ambhorkar P. Wang Z. Ko H. Lee S. Koo K. I. Kim K.. (2018). Nanowire-based biosensors: from growth to applications. Micromachines 9:679. 10.3390/mi9120679 30572645 PMC6316191

[B3] Anaraki E. H. Kermanpur A. Steier L. Domanski K. Matsui T. Tress W. (2016). Highly efficient and stable planar perovskite solar cells by solution-processed tin oxide. Energy Environ. Sci. 9, 3128–3134. 10.1039/C6EE02390H

[B4] Bao Q. Li W. Xu P. Zhang M. Dai D. Wang P.. (2020). On-chip single-mode CdS nanowire laser. Light Sci. Appl. 9:42. 10.1038/s41377-020-0277-0 32194956 PMC7073330

[B5] Barnes W. L. Dereux A. Ebbesen T. W. (2003). Surface plasmon subwavelength optics. Nature 424, 824–830. 10.1038/nature01937 12917696

[B6] Bawendi M. G. Steigerwald M. L. Brus L. E. (1990). The quantum mechanics of larger semiconductor clusters (“Quantum Dots”). Annu. Rev. Phys. Chem. 41, 477–496. 10.1146/annurev.pc.41.100190.002401

[B7] Bi D. Tress W. Dar M. I. Gao P. Luo J. Renevier C.. (2016). Efficient luminescent solar cells based on tailored mixed-cation perovskites. Sci. Adv. 2:e1501170. 10.1126/sciadv.1501170 26767196 PMC4705040

[B8] Björk M. T. Ohlsson B. J. Sass T. Persson A. I. Thelander C. Magnusson M. H. (2002). One-dimensional heterostructures in semiconductor nanowhiskers. Appl. Phys. Lett. 80, 1058–1060. 10.1063/1.1447312

[B9] Brenner T. M. Egger D. A. Kronik L. Hodes G. Cahen D. (2016). Hybrid organic—inorganic perovskites: low-cost semiconductors with intriguing charge-transport properties. Nat. Rev. Mater. 1:15007 10.1038/natrevmats.2015.7

[B10] Burstein E. (1954). Anomalous optical absorption limit in InSb. Phys. Rev. 93, 632–633. 10.1103/PhysRev.93.632

[B11] Chen B. Wu H. Xin C. Dai D. Tong L. (2017). Flexible integration of free-standing nanowires into silicon photonics. Nat. Commun. 8:20. 10.1038/s41467-017-00038-0 28615617 PMC5471269

[B12] Chen R. T, Tran, T. D. Ng K. W. Ko W. S. Chuang L. C. Sedgwick F. G. (2011). Nanolasers grown on silicon. Nat. Photonics 5, 170–175. 10.1038/nphoton.2010.315

[B13] Cheon S. E. Lee H. S. Choi J. Jeong A. R. Lee T. S. Jeong D. S.. (2017). Fabrication of parabolic Si nanostructures by nanosphere lithography and its application for solar cells. Sci. Rep. 7:7336. 10.1038/s41598-017-07463-7 28779077 PMC5544770

[B14] Chin A. H. Vaddiraju S. Maslov A. V. Ning C. Z. Sunkara M. K. Meyyappan M. (2006). Near-infrared semiconductor subwavelength-wire lasers. Appl. Phys. Lett. 88:163115 10.1063/1.2198017

[B15] Cho D. Park J. Kim T. Jeon S. (2019). Recent advances in lithographic fabrication of micro-/nanostructured polydimethylsiloxanes and their soft electronic applications. J. Semiconduct. 40:111605 10.1088/1674-4926/40/11/111605

[B16] Chou Y. H. Chou B. T. Chiang C. K. Lai Y. Y. Yang C. T. Li H.. (2015). Ultrastrong mode confinement in ZnO surface plasmon nanolasers. ACS Nano 9, 3978–3983. 10.1021/acsnano.5b01643 25853853

[B17] Chou Y. H. Wu Y. M. Hong K. B. Chou B. T. Shih J. H. Chung Y. C.. (2016). High-operation-temperature plasmonic nanolasers on single-crystalline aluminum. Nano Lett. 16, 3179–3186. 10.1021/acs.nanolett.6b00537 27089144

[B18] Chu T. Fujioka N. Ishizaka M. (2009). Compact, lower-power-consumption wavelength tunable laser fabricated with silicon photonic-wire waveguide micro-ring resonators. Opt. Express 17, 14063–14068. 10.1364/OE.17.014063 19654814

[B19] Colli A. Fasoli A. Beecher P. Servati P. Pisana S. Fu Y. (2007). Thermal and chemical vapor deposition of Si nanowires: shape control, dispersion, electrical properties. J. Appl. Phys. 102:034302 10.1063/1.2764050

[B20] Correa-Baena J. P. Saliba M. Buonassisi T. Gratzel M. Abate A. Tress W.. (2017). Promises and challenges of perovskite solar cells. Science 358, 739–744. 10.1126/science.aam6323 29123060

[B21] Cui Y. Duan X. Hu J. Lieber C. M. (2000). Doping and electrical transport in silicon nanowires. J. Phys. Chem. B 104, 5213–5216. 10.1021/jp0009305

[B22] Dang C. Lee J. Breen C. Steckel J. S. Coe-Sullivan S. Nurmikko A. (2012). Red, green and blue lasing enabled by single-exciton gain in colloidal quantum dot films. Nat. Nanotechnol. 7, 335–339. 10.1038/nnano.2012.61 22543426

[B23] Dasgupta N. P. Sun J. Liu C. Brittman S. Andrews S. C. Lim J.. (2014). 25th anniversary article: semiconductor nanowires–synthesis, characterization, and applications. Adv. Mater. 26, 2137–2184. 10.1002/adma.201305929 24604701

[B24] De Leon I. Berini P. (2010). Amplification of long-range surface plasmons by a dipolar gain medium. Nat. Photonics 4, 382–387. 10.1038/nphoton.2010.37

[B25] Deschler F. Price M. Pathak S. Klintberg L. E. Jarausch D. D. Higler R. (2014). High photoluminescence efficiency and optically pumped lasing in solution-processed mixed halide perovskite semiconductors. J. Phys. Chem. Lett. 5, 1421–1426. 10.1021/jz5005285 26269988

[B26] Ding J. X. Zapien J. A. Chen W. W. Lifshitz Y. Lee S. T. Meng X. M. (2004). Lasing in ZnS nanowires grown on anodic aluminum oxide templates. Appl. Phys. Lett. 85, 2361–2363. 10.1063/1.1791326

[B27] Ding K. Hill M. T. Liu Z. C. Yin L. J. van Veldhoven P. J. Ning C. Z. (2013). Record performance of electrical injection sub-wavelength metallic-cavity semiconductor lasers at room temperature. Opt. Express 21, 4728–4733. 10.1364/OE.21.004728 23482005

[B28] Djurišić A. B. Ng A. M. C. Chen X. Y. (2010). ZnO nanostructures for optoelectronics: material properties and device applications. Progr. Quantum Electron. 34, 191–259. 10.1016/j.pquantelec.2010.04.001

[B29] Duan X. Huang Y. Agarwal R. Lieber C. M. (2003). Single-nanowire electrically driven lasers. Nature 421, 241–245. 10.1038/nature01353 12529637

[B30] Duan X. Huang Y. Cui Y. Wang J. Lieber C. M. (2001). Indium phosphide nanowires as building blocks for nanoscale electronic and optoelectronic devices. Nature 409, 66–69. 10.1038/35051047 11343112

[B31] Duan X. Lieber C. M. (2000a). General synthesis of compound semiconductor nanowires. Adv. Mater. 12, 298–302. 10.1002/(SICI)1521-4095(200002)12:4<298::AID-ADMA298>3.0.CO;2-Y

[B32] Duan X. Lieber C. M. (2000b). Laser-assisted catalytic growth of single crystal gan nanowires. J. Am. Chem. Soc. 122, 188–189. 10.1021/ja993713u

[B33] Eaton S. W. Fu A. Wong A. B. Ning C.-Z. Yang P. (2016). Semiconductor nanowire lasers. Nat. Rev. Mater. 1:16028 10.1038/natrevmats.2016.28

[B34] Gao H. Fu A. Andrews S. C. Yang P. (2013). Cleaved-coupled nanowire lasers. Proc. Natl. Acad. Sci. U.S.A. 110, 865–869. 10.1073/pnas.1217335110 23284173 PMC3549097

[B35] Gao P. Pu M. Ma X. Li X. Guo Y. Wang C.. (2020). Plasmonic lithography for the fabrication of surface nanostructures with a feature size down to 9 nm. Nanoscale 12, 2415–2421. 10.1039/C9NR08153D 31750491

[B36] Gatdula R. Abbaslou S. Lu M. Stein A. Jiang W. (2019). Guiding light in bent waveguide superlattices with low crosstalk. Optica 6, 585–591. 10.1364/OPTICA.6.000585 25960367

[B37] Gather M. C. Meerholz K. Danz N. Leosson K. (2010). Net optical gain in a plasmonic waveguide embedded in a fluorescent polymer. Nat. Photonics 4, 457–461. 10.1038/nphoton.2010.121

[B38] Gradečak S. Qian F. Li Y. Park H.-G. Lieber C. M. (2005). GaN nanowire lasers with low lasing thresholds. Appl. Phys. Lett. 87:173111 10.1063/1.2115087

[B39] Green M. A. Ho-Baillie A. Snaith H. J. (2014). The emergence of perovskite solar cells. Nat. Photonics 8, 506–514. 10.1038/nphoton.2014.134

[B40] Gu F. Yang Z. Yu H. Xu J. Wang P. Tong L.. (2011). Spatial bandgap engineering along single alloy nanowires. J. Am. Chem. Soc. 133, 2037–2039. 10.1021/ja110092a 21271702

[B41] Gu F. Zhang L. Yin X. Tong L. (2008). Polymer single-nanowire optical sensors. Nano Lett. 8, 2757–2761. 10.1021/nl8012314 18672942

[B42] Gu Z. Liu S. Sun S. Wang K. Lyu Q. Xiao S. (2015). Photon hopping and nanowire based hybrid plasmonic waveguide and ring-resonator. Sci. Rep. 5:9171 10.1038/srep09171

[B43] Gu Z. Sun W. Wang K. Zhang N. Zhang C. Lyu Q. (2016). Hybridizing CH3NH3PbBr3 microwires and tapered fibers for efficient light collection. J. Mater. Chem. A 4, 8015–8019. 10.1039/C6TA01620K

[B44] Guo X. Qiu M. Bao J. Wiley B. J. Yang Q. Zhang X.. (2009). Direct coupling of plasmonic and photonic nanowires for hybrid nanophotonic components and circuits. Nano Lett. 9, 4515–4519. 10.1021/nl902860d 19995088

[B45] Gupta S. Navaraj W. T. Lorenzelli L. Dahiya R. (2018). Ultra-thin chips for high-performance flexible electronics. NPJ Flexible Electronics. 2:8 10.1038/s41528-018-0021-5

[B46] Ha S. T. Su R. Xing J. Zhang Q. Xiong Q. (2017). Metal halide perovskite nanomaterials: synthesis and applications. Chem. Sci. 8, 2522–2536. 10.1039/C6SC04474C 28553484 PMC5431666

[B47] Harter T. Muehlbrandt S. Ummethala S. Schmid A. Nellen S. Hahn L. (2018). Silicon–plasmonic integrated circuits for terahertz signal generation and coherent detection. Nat. Photonics 12, 625–633. 10.1038/s41566-018-0237-x

[B48] He R. Gao D. Fan R. Hochbaum A. I. Carraro C. Maboudian R. (2005). Si nanowire bridges in microtrenches: integration of growth into device fabrication. Adv. Mater. 17, 2098–2102. 10.1002/adma.200401959

[B49] Heo Y. W. Norton D. P. Tien L. C. Kwon Y. Kang B. S. Ren F. (2004). ZnO nanowire growth and devices. Mater. Sci. Eng. R 47, 1–47. 10.1016/j.mser.2004.09.001

[B50] Hill M. T. Oei Y.-S. Smalbrugge B. Y. Zhu de Vries T. van Veldhoven P. J. (2007). Lasing in metallic-coated nanocavities. Nat. Photonics 1, 589–594. 10.1038/nphoton.2007.171

[B51] Hinkley E. D. Kelley P. L. (1971). Detection of air pollutants with tunable diode lasers. Science 171, 635–639. 10.1126/science.171.3972.635 5540302

[B52] Hochbaum A. I. Fan R. He R. Yang P. (2005). Controlled growth of Si nanowire arrays for device integration. Nano Lett. 5, 457–460. 10.1021/nl047990x 15755094

[B53] Hsu Y. J. Lu S. Y. (2005). Low temperature growth and dimension-dependent photoluminescence efficiency of semiconductor nanowires. Appl. Phys. Mater. Sci. Process. 81, 573–578. 10.1007/s00339-004-2714-y

[B54] Huang J. Yuan Y. Shao Y. Yan Y. (2017). Understanding the physical properties of hybrid perovskites for photovoltaic applications. Nat. Rev. Mater. 2:17042 10.1038/natrevmats.2017.42

[B55] Huang M. H. Mao S. Feick H. Yan H. Wu Y. Kind H.. (2001). Room-temperature ultraviolet nanowire nanolasers. Science 292, 1897–1899. 10.1126/science.1060367 11397941

[B56] Jaramillo-Quintero O. A. Sanchez R. S. Rincon M. Mora-Sero I. (2015). Bright visible-infrared light emitting diodes based on hybrid halide perovskite with spiro-OMeTAD as a hole-injecting layer. J. Phys. Chem. Lett. 6, 1883–1890. 10.1021/acs.jpclett.5b00732 26263264

[B57] Johansson J. Karlsson L. S. Svensson C. P. Martensson T. Wacaser B. A. Deppert K.. (2006). Structural properties of <111>B -oriented III-V nanowires. Nat. Mater. 5, 574–580. 10.1038/nmat1677 16783358

[B58] Johnson J. C. Choi H. J. Knutsen K. P. Schaller R. D. Yang P. Saykally R. J. (2002). Single gallium nitride nanowire lasers. Nat. Mater. 1, 106–110. 10.1038/nmat728 12618824

[B59] Johnson J. C. Yan H. Schaller R. D. Haber L. H. Saykally R. J. Yang P. (2001). Single nanowire lasers. J. Phys. Chem. B 105, 11387–11390. 10.1021/jp012304t

[B60] Johnson J. C. Yan H. Yang P. Saykally R. J. (2003). Optical cavity effects in ZnO nanowire lasers and waveguides. J. Phys. Chem. B 107, 8816–8828. 10.1021/jp034482n

[B61] Katagiri Y. Takahara J. Kobayashi T. (2004). Nano-optical waveguides breaking through diffraction limit of light. Optomechatr. Micro Nano Components Dev. Syst. Proc. SPIE 5604:158 10.1117/12.582740

[B62] Kim H. S. Lee C. R. Im J. H. Lee K. B. Moehl T. Marchioro A.. (2012). Lead iodide perovskite sensitized all-solid-state submicron thin film mesoscopic solar cell with efficiency exceeding 9%. Sci. Rep. 2:591. 10.1038/srep00591 22912919 PMC3423636

[B63] Kuykendall T. Pauzauskie P. J. Zhang Y. Goldberger J. Sirbuly D. Denlinger J.. (2004). Crystallographic alignment of high-density gallium nitride nanowire arrays. Nat. Mater. 3, 524–528. 10.1038/nmat1177 15273744

[B64] Kuykendall T. Ulrich P. Aloni S. Yang P. (2007). Complete composition tunability of InGaN nanowires using a combinatorial approach. Nat. Mater. 6, 951–956. 10.1038/nmat2037 17965718

[B65] Law M. Sirbuly D. J. Johnson J. C. Goldberger J. Saykally R. J. Yang P. (2004). Nanoribbon waveguides for subwavelength photonics integration. Science 305, 1269–1273. 10.1126/science.1100999 15333835

[B66] Li J. Meng C. Liu Y. Wu X. Lu Y. Ye Y.. (2013). Wavelength tunable CdSe nanowire lasers based on the absorption-emission-absorption process. Adv. Mater. 25, 833–7. 10.1002/adma.201203692 23135956

[B67] Li Q. Wright J. B. Chow W. W. Luk T. S. Brener I. Lester L. F.. (2012). Single-mode GaN nanowire lasers. Opt. Express 20, 17873–17879. 10.1364/OE.20.017873 23038337

[B68] Liu N. Wei H. Li J. Wang Z. Tian X. Pan A.. (2013). Plasmonic amplification with ultra-high optical gain at room temperature. Sci. Rep. 3:1967. 10.1038/srep01967 23752666 PMC3678133

[B69] Liu X. Zhang Q. Yip J. N. Xiong Q. Sum T. C. (2013). Wavelength tunable single nanowire lasers based on surface plasmon polariton enhanced burstein-moss effect. Nano Lett. 13, 5336–5343. 10.1021/nl402836x 24134588

[B70] Liu Y. Zapien J. A. Shan Y. Y. Geng C. Y. Lee C. S. Lee S. T. (2005). Wavelength-controlled lasing in ZnxCd1-xS single-crystal nanoribbons. Adv. Mater. 17, 1372–1377. 10.1002/adma.200401606 34412431

[B71] Liu Y. K. Zapien J. A. Shan Y. Y. Tang H. Lee C. S. Lee S. T. (2007). Wavelength-tunable lasing in single-crystal CdS1–XSeX nanoribbons. Nanotechnology 18:365606 10.1088/0957-4484/18/36/365606

[B72] Liu Z. Yin L. Ning H. Yang Z. Tong L. Ning C. Z. (2013). Dynamical color-controllable lasing with extremely wide tuning range from red to green in a single alloy nanowire using nanoscale manipulation. Nano Lett. 13, 4945–4950. 10.1021/nl4029686 24016196

[B73] Lu Y. J. Kim J. Chen H. Y. Wu C. Dabidian N. Sanders C. E.. (2012). Plasmonic nanolaser using epitaxially grown silver film. Science 337, 450–453. 10.1126/science.1223504 22837524

[B74] Lu Y. J. Wang C. Y. Kim J. Chen H. Y. Lu M. Y. Chen Y. C.. (2014). All-color plasmonic nanolasers with ultralow thresholds: autotuning mechanism for single-mode lasing. Nano Lett. 14, 4381–4388. 10.1021/nl501273u 25029207

[B75] Luo L. W. Ophir N. Chen C. P. Gabrielli L. H. Poitras C. B. Bergmen K.. (2014). WDM-compatible mode-division multiplexing on a silicon chip. Nat. Commun. 5:3069. 10.1038/ncomms4069 24423882

[B76] Mårtensson T. Wagner J. B. Hilner E. Mikkelsen A. Thelander C. Stangl J. (2007). Epitaxial growth of indium arsenide nanowires on silicon using nucleation templates formed by self-assembled organic coatings. Adv. Mater. 19, 1801–1806. 10.1002/adma.200700285

[B77] Ma R. M. Oulton R. F. Sorger V. J. Bartal G. Zhang X. (2011). Room-temperature sub-diffraction-limited plasmon laser by total internal reflection. Nat. Mater. 10, 110–113. 10.1038/nmat2919 21170028

[B78] Ma R. M. Wei X. L. Dai L. Liu S. F. Chen T. Yue S.. (2009). Light coupling and modulation in coupled nanowire ring-fabry-perot cavity. Nano Lett. 9, 2697–2703. 10.1021/nl901190v 19534464

[B79] Ma R. M. Yin X. Oulton R. F. Sorger V. J. Zhang X. (2012). Multiplexed and electrically modulated plasmon laser circuit. Nano Lett. 12, 5396–5402. 10.1021/nl302809a 22989288

[B80] Ma Y. Guo X. Wu X. Dai L. Tong L. (2013). Semiconductor nanowire lasers. Adv. Opt. Photonics 5:216. 10.1364/AOP.5.00021626146369

[B81] Maier S. A. Kik P. G. Atwater H. A. Meltzer S. Harel E. Koel B. E.. (2003). Local detection of electromagnetic energy transport below the diffraction limit in metal nanoparticle plasmon waveguides. Nat. Mater. 2, 229–232. 10.1038/nmat852 12690394

[B82] Maslov A. V. Ning C. Z. (2004). Modal gain in a semiconductor nanowire laser with anisotropic bandstructure. IEEE J. Quantum Electron. 40, 1389–1397. 10.1109/JQE.2004.834767

[B83] Meng C. Xiao Y. Wang P. Zhang L. Liu Y. Tong L. (2011). Quantum-dot-doped polymer nanofibers for optical sensing. Adv. Mater. 23, 3770–3774. 10.1002/adma.201101392 21766349

[B84] Moore D. Wang Z. L. (2006). Growth of anisotropic one-dimensional ZnS nanostructures. J. Mater. Chem. 16, 3898–3905. 10.1039/b607902b

[B85] Morales A. M. Lieber C. M. (1998). A laser ablation method for the synthesis of crystalline semiconductor nanowires. Science 279, 208–211. 10.1126/science.279.5348.208 9422689

[B86] Mrejen M. Suchowski H. Hatakeyama T. Wu C. Feng L. O'Brien K.. (2015). Adiabatic elimination-based coupling control in densely packed subwavelength waveguides. Nat. Commun. 6:7565. 10.1038/ncomms8565 26113179 PMC4491806

[B87] Neumann A. Wierer J. J. Jr. Davis W. Ohno Y. Brueck S. R. Tsao J. Y. (2011). Four-color laser white illuminant demonstrating high color-rendering quality. Opt. Express 19(Suppl. 4), A982–A990. 10.1364/OE.19.00A982 21747570

[B88] Nezhad M. P. Simic A. Bondarenko O. Slutsky B. Mizrahi A. Feng L.. (2010). Room-temperature subwavelength metallo-dielectric lasers. Nat. Photonics 4, 395–399. 10.1038/nphoton.2010.8822109001

[B89] Noborisaka J. Motohisa J. Fukui T. (2005). Catalyst-free growth of GaAs nanowires by selective-area metalorganic vapor-phase epitaxy. Appl. Phys. Lett. 86:213102. 10.1063/1.193503829300185

[B90] Noginov M. A. Zhu G. Belgrave A. M. Bakker R. Shalaev V. M. Narimanov E. E.. (2009). Demonstration of a spaser-based nanolaser. Nature 460, 1110–1112. 10.1038/nature08318 19684572

[B91] Oulton R. F. Sorger V. J. Genov D. A. Pile D. F. P. Zhang X. (2008). A hybrid plasmonic waveguide for subwavelength confinement and long-range propagation. Nat. Photonics 2, 496–500. 10.1038/nphoton.2008.131

[B92] Oulton R. F. Sorger V. J. Zentgraf T. Ma R. M. Gladden C. Dai L.. (2009). Plasmon lasers at deep subwavelength scale. Nature 461, 629–632. 10.1038/nature08364 19718019

[B93] Pan A. Liu R. Sun M. Ning C. Z. (2009a). Quaternary alloy semiconductor nanobelts with bandgap spanning the entire visible spectrum. J. Am. Chem. Soc. 131, 9502–9503. 10.1021/ja904137m 19545159

[B94] Pan A. Liu R. Wang F. Xie S. Zou B. Zacharias M.. (2006). High-quality alloyed CdSxSe1-x whiskers as waveguides with tunable stimulated emission. J. Phys. Chem. B 110, 22313–22317. 10.1021/jp064664s 17091969

[B95] Pan A. Yang H. Liu R. Yu R. Zou B. Wang Z. (2005). Color-tunable photoluminescence of alloyed CdS(x)Se(1-x) nanobelts. J. Am. Chem. Soc. 127, 15692–15693. 10.1021/ja056116i 16277497

[B96] Pan A. Zhou W. Leong E. S. Liu R. Chin A. H. Zou B.. (2009b). Continuous alloy-composition spatial grading and superbroad wavelength-tunable nanowire lasers on a single chip. Nano Lett. 9, 784–788. 10.1021/nl803456k 19173627

[B97] Park H. Crozier K. B. (2013). Multispectral imaging with vertical silicon nanowires. Sci. Rep. 3:2460. 10.1038/srep02460 23955156 PMC3746203

[B98] Pauzauskie P. J. Sirbuly D. J. Yang P. (2006). Semiconductor nanowire ring resonator laser. Phys. Rev. Lett. 96:143903. 10.1103/PhysRevLett.96.143903 16712076

[B99] Pigtailed C. D. S. (2010). Nanoribbon ring laser. Appl. Phys. Lett. 97:153122 10.1063/1.3501969

[B100] Radovanovic P. V. Barrelet C. J. Gradecak S. Qian F. Lieber C. M. (2005). General synthesis of manganese-doped II-VI and III-V semiconductor nanowires. Nano Lett. 5, 1407–1411. 10.1021/nl050747t 16178248

[B101] Rahimi F. Jafari A. K. Hsu C. A. Ferekides C. S. Hoff A. M. (2019). Selective sensing in perovskite-based image sensors. Organ. Electron. 75:105397 10.1016/j.orgel.2019.105397

[B102] Saidaminov M. I. Adinolfi V. Comin R. Abdelhady A. L. Peng W. Dursun I.. (2015). Planar-integrated single-crystalline perovskite photodetectors. Nat. Commun. 6:8724. 10.1038/ncomms9724 26548941 PMC4667636

[B103] Saxena D. Mokkapati S. Parkinson P. Jiang N. Gao Q. Tan H. H.. (2013). Optically pumped room-temperature GaAs nanowire lasers. Nat. Photonics 7, 963–968. 10.1038/nphoton.2013.30327459233

[B104] Seo M. K. Yang J. K. Jeong K. Y. Park H. G. Qian F. Ee H. S.. (2008). Modal characteristics in a single-nanowire cavity with a triangular cross section. Nano Lett. 8, 4534–4538. 10.1021/nl8027125 19367886

[B105] Shtrikman H. Popovitz-Biro R. Kretinin A. Heiblum M. (2009). Stacking-faults-free zinc blende GaAs nanowires. Nano Lett. 9, 215–219. 10.1021/nl8027872 19093840

[B106] Sidiropoulos T. P. H. Röder R. Geburt S. Hess O. Maier S. A. Ronning C. (2014). Ultrafast plasmonic nanowire lasers near the surface plasmon frequency. Nat. Phys. 10, 870–876. 10.1038/nphys3103

[B107] Silveirinha M. Engheta N. (2006). Tunneling of electromagnetic energy through subwavelength channels and bends using epsilon-near-zero materials. Phys. Rev. Lett. 97:157403. 10.1103/PhysRevLett.97.157403 17155357

[B108] Sirbuly D. J. Law M. Pauzauskie P. Yan H. Maslov A. V. Knutsen K.. (2005). Optical routing and sensing with nanowire assemblies. Proc. Natl. Acad. Sci. U.S.A. 102, 7800–7805. 10.1073/pnas.0408641102 15911765 PMC1142354

[B109] Sivakov V. Andrä G. Himcinschi C. Gösele U. Zahn D. R. T. Christiansen S. (2006). Growth peculiarities during vapor–liquid–solid growth of silicon nanowhiskers by electron-beam evaporation. Appl. Phys. A 85, 311–315. 10.1007/s00339-006-3675-0

[B110] Song Q. H. Cao H. (2010). Improving optical confinement in nanostructures via external mode coupling. Phys. Rev. Lett. 105:053902. 10.1103/PhysRevLett.105.053902 20867919

[B111] Stockman M. I. (2010). The spaser as a nanoscale quantum generator and ultrafast amplifier. J. Opt. 12:024004 10.1088/2040-8978/12/2/024004

[B112] Stranks S. D. Snaith H. J. (2015). Metal-halide perovskites for photovoltaic and light-emitting devices. Nat. Nanotechnol. 10, 391–402. 10.1038/nnano.2015.90 25947963

[B113] Sum T. C. Mathews N. (2014). Advancements in perovskite solar cells: photophysics behind the photovoltaics. Energy Environ. Sci. 7, 2518–2534. 10.1039/C4EE00673A

[B114] Sun Q. C. Yadgarov L. Rosentsveig R. Seifert G. Tenne R. Musfeldt J. L. (2013). Observation of a burstein-moss shift in rhenium-doped MoS2 nanoparticles. ACS Nano 7, 3506–3511. 10.1021/nn400464g 23477349

[B115] Sutherland B. R. Hoogland S. Adachi M. M. Wong C. T. Sargent E. H. (2014). Conformal organohalide perovskites enable lasing on spherical resonators. ACS Nano 8, 10947–10952. 10.1021/nn504856g 25313937

[B116] Takahara J. Yamagishi S. Taki H. Morimoto A. Kobayashi T. (1997). Guiding of a one-dimensional optical beam with nanometer diameter. Opt. Lett. 22, 475–477. 10.1364/OL.22.000475 18183239

[B117] Tan Z. K. Moghaddam R. S. Lai M. L. Docampo P. Higler R. Deschler F.. (2014). Bright light-emitting diodes based on organometal halide perovskite. Nat. Nanotechnol. 9, 687–692. 10.1038/nnano.2014.149 25086602

[B118] Thomas D. G. Hopfield J. J. (1962). Optical properties of bound exciton complexes in cadmium sulfide. Phys. Rev. 128, 2135–2148. 10.1103/PhysRev.128.2135

[B119] Venugopal R. Lin P. I. Liu C. C. Chen Y. T. (2005). Surface-enhanced Raman scattering and polarized photoluminescence from catalytically grown CdSe nanobelts and sheets. J. Am. Chem. Soc. 127, 11262–11268. 10.1021/ja044270j 16089453

[B120] Wagner R. S. Ellis W. C. (1964). Vapor-liquid-solid mechanism of single crystal growth. Appl. Phys. Lett. 4, 89–90. 10.1063/1.1753975

[B121] Wang G. Jiang X. Zhao M. Ma Y. Fan H. Yang Q.. (2012). Microlaser based on a hybrid structure of a semiconductor nanowire and a silica microdisk cavity. Opt. Express 20, 29472–29478. 10.1364/OE.20.029472 23388773

[B122] Wang K. Gu Z. Liu S. Li J. Xiao S. Song Q. (2016). Formation of single-mode laser in transverse plane of perovskite microwire via micromanipulation. Opt. Lett. 41, 555–558. 10.1364/OL.41.000555 26907422

[B123] Wang S. Wang K. Gu Z. Wang Y. Huang C. Yi N. (2017). Solution-phase synthesis of cesium lead halide perovskite microrods for high-quality microlasers and photodetectors. Adv. Opti. Mater. 5:1700023 10.1002/adom.201700023

[B124] Wu C. Y. Kuo C. T. Wang C. Y. He C. L. Lin M. H. Ahn H.. (2011). Plasmonic green nanolaser based on a metal-oxide-semiconductor structure. Nano Lett. 11, 4256–4260. 10.1021/nl2022477 21882819

[B125] Wu X. Xiao Y. Meng C. Zhang X. Yu S. Wang Y.. (2013). Hybrid photon-plasmon nanowire lasers. Nano Lett. 13, 5654–5659. 10.1021/nl403325j 24144390

[B126] Wu Y. Yang P. (2000). Germanium nanowire growth via simple vapor transport. Chem. Mater. 12, 605–607. 10.1021/cm9907514

[B127] Wu Y. Yang P. (2001). Direct observation of vapor–liquid–solid nanowire growth. J. Am. Chem. Soc. 123, 3165–3166. 10.1021/ja0059084

[B128] Xia Y. Yang P. Sun Y. Wu Y. Mayers B. Gates B. (2003). One-dimensional nanostructures: synthesis, characterization, and applications. Adv. Mater. 15, 353–389. 10.1002/adma.200390087

[B129] Xiao Y. Meng C. Wang P. Ye Y. Yu H. Wang S.. (2011). Single-nanowire single-mode laser. Nano Lett. 11, 1122–1126. 10.1021/nl1040308 21322600

[B130] Xing J. Liu X. F. Zhang Q. Ha S. T. Yuan Y. W. Shen C.. (2015). Vapor phase synthesis of organometal halide perovskite nanowires for tunable room-temperature nanolasers. Nano Lett. 15, 4571–4577. 10.1021/acs.nanolett.5b01166 26043362

[B131] Yan R. Pausauskie P. Huang J. Yang P. (2009). Direct photonic-plasmonic coupling and routing in single nanowires. Proc. Natl. Acad. Sci. U.S.A. 106, 21045–21050. 10.1073/pnas.0902064106 19955430 PMC2795563

[B132] Yang P. Lieber C. M. (1996). Nanorod-superconductor composites: a pathway to materials with high critical current densities. Science 273, 1836–1840. 10.1126/science.273.5283.1836

[B133] Yang P. Yan H. Mao S. Russo R. Johnson J. Saykally R. (2002). Controlled growth of ZnO nanowires and their optical properties. Adv. Func. Mater. 12, 323–331. 10.1002/1616-3028(20020517)12:5<323::AID-ADFM323>3.0.CO;2-G

[B134] Yang W. S. Noh J. H. Jeon N. J. Kim Y. C. Ryu S. Seo J.. (2015). SOLAR CELLS. High-performance photovoltaic perovskite layers fabricated through intramolecular exchange. Science 348, 1234–1237. 10.1126/science.aaa9272 25999372

[B135] Yang X. Ni P. N. Jing P. T. Zhang L. G. Ma R. M. Shan C. X. (2019). Room temperature electrically driven ultraviolet plasmonic lasers. Adv. Opt. Mater. 7:1801681 10.1002/adom.201801681

[B136] Yang Z. Albrow-Owen T. Cui H. Alexander-Webber J. Gu F. Wang X.. (2019). Single-nanowire spectrometers. Science. 365, 1017–1020. 10.1126/science.aax8814 31488686

[B137] Yang Z. Xu J. Wang P. Zhuang X. Pan A. Tong L. (2011). On-nanowire spatial band gap design for white light emission. Nano Lett. 11, 5085–5089. 10.1021/nl203529h 22011228

[B138] Yazawa M. Koguchi M. Hiruma K. (1991). Heteroepitaxial ultrafine wire-like growth of InAs on GaAs substrates. Appl. Phys. Lett. 58, 1080–1082. 10.1063/1.104377

[B139] Zapien J. A. Liu Y. K. Shan Y. Y. Tang H. Lee C. S. Lee S. T. (2007). Continuous near-infrared-to-ultraviolet lasing from II-VI nanoribbons. Appl. Phys. Lett. 90:213114 10.1063/1.2736286

[B140] Zhai T. Fang X. Li L. Bando Y. Golberg D. (2010). One-dimensional CdS nanostructures: synthesis, properties, and applications. Nanoscale 2, 168–187. 10.1039/b9nr00415g 20644793

[B141] Zhang C. Zhang F. Xia T. Kumar N. Hahm J. I. Liu J.. (2009). Low-threshold two-photon pumped ZnO nanowire lasers. Opt Express 17, 7893–7900. 10.1364/OE.17.007893 19434120

[B142] Zhang C. Zou C. L. Dong H. Yan Y. Yao J. Zhao Y. S. (2017). Dual-color single-mode lasing in axially coupled organic nanowire resonators. Sci. Adv. 3:e1700225. 10.1126/sciadv.1700225 28785731 PMC5524526

[B143] Zhang C. Zou C. L. Zhao Y. Dong C. H. Wei C. Wang H.. (2015). Organic printed photonics: from microring lasers to integrated circuits. Sci. Adv. 1:e1500257. 10.1126/sciadv.1500257 26601256 PMC4643768

[B144] Zhang C. F. Dong Z. W. You G. J. Qian S. X. Deng H. (2006). Multiphoton route to ZnO nanowire lasers. Opt. Lett. 31, 3345–3347. 10.1364/OL.31.003345 17072418

[B145] Zhang J. Shokouhi B. Cui B. (2012). Tilted nanostructure fabrication by electron beam lithography. J. Vacuum Sci. Technol. B Nanotechnol. Microelectr. 30:06F302 10.1116/1.4754809

[B146] Zhang J. Zhang L. D. Wang X. F. Liang C. H. Peng X. S. Wang Y. W. (2001). Fabrication and photoluminescence of ordered GaN nanowire arrays. J. Chem. Phys. 115, 5714–5717. 10.1063/1.1407005

[B147] Zhang N. Fan Y. Wang K. Gu Z. Wang Y. Ge L.. (2019). All-optical control of lead halide perovskite microlasers. Nat. Commun. 10:1770. 10.1038/s41467-019-09876-6 30992442 PMC6467983

[B148] Zhang Q. Ha S. T. Liu X. Sum T. C. Xiong Q. (2014a). Room-temperature near-infrared high-Q perovskite whispering-gallery planar nanolasers. Nano Lett. 14, 5995–6001. 10.1021/nl503057g 25118830

[B149] Zhang Q. Li G. Liu X. Qian F. Li Y. Sum T. C.. (2014b). A room temperature low-threshold ultraviolet plasmonic nanolaser. Nat. Commun. 5:4953. 10.1038/ncomms5953 25247634

[B150] Zhang Q. Su R. Du W. Liu X. Zhao L. Ha S. T. (2017). Advances in small perovskite-based lasers. Small Methods. 1:1700163 10.1002/smtd.201700163

[B151] Zhang Y. Russo R. E. Mao S. S. (2005). Quantum efficiency of ZnO nanowire nanolasers. Appl. Phys. Lett. 87:043106 10.1063/1.2001754

[B152] Zhang Y. Saxena D. Aagesen M. Liu H. (2019). Toward electrically driven semiconductor nanowire lasers. Nanotechnology 30:192002. 10.1088/1361-6528/ab000d 30658345

[B153] Zhao J. Yan Y. Wei C. Zhang W. Gao Z. Zhao Y. S. (2018). Switchable single-mode perovskite microlasers modulated by responsive organic microdisks. Nano Lett. 18, 1241–1245. 10.1021/acs.nanolett.7b04834 29323922

[B154] Zhao L. J. Hu L. F. Fang X. S. (2012). Growth and device application of cdse nanostructures. Adv. Funct. Mater. 22, 1551–1566. 10.1002/adfm.201103088

[B155] Zhou W. Dridi M. Suh J. Y. Kim C. H. Co D. T. Wasielewski M. R.. (2013). Lasing action in strongly coupled plasmonic nanocavity arrays. Nat. Nanotechnol. 8, 506–511. 10.1038/nnano.2013.99 23770807

[B156] Zhu H. Fu Y. Meng F. Wu X. Gong Z. Ding Q.. (2015). Lead halide perovskite nanowire lasers with low lasing thresholds and high quality factors. Nat. Mater. 14, 636–642. 10.1038/nmat4271 25849532

[B157] Zhu Z. Sun Q. Zhang Z. Dai J. Xing G. Li S. (2018). Metal halide perovskites: stability and sensing-ability. J. Mater. Chem. C 6, 10121–10137. 10.1039/C8TC03164A

